# Kinetics, central composite design and artificial neural network modelling of ciprofloxacin antibiotic photodegradation using fabricated cobalt-doped zinc oxide nanoparticles

**DOI:** 10.1038/s41598-024-84568-w

**Published:** 2025-01-10

**Authors:** Asmaa I. Meky, Mohamed A. Hassaan, Mohamed A. El-Nemr, Howida A. Fetouh, Amel M. Ismail, Ahmed El Nemr

**Affiliations:** 1 Department of Chemistry, Faculty of Science, Alexandria University, Alexandria, Egypt; 2 Environment Division, National Institute of Oceanography and Fisheries (NIOF), Kayet Bey, Elanfoushy, Alexandria, Egypt; 3 Department of Chemical Engineering, Faculty of Engineering, Minia University, Minia, 61519 Egypt

**Keywords:** Central composite design, Ciprofloxacin, Co-ZnO NPs, Co-precipitation, Photocatalysis, Wastewater treatment, Chemical engineering, Environmental chemistry

## Abstract

Cobalt-doped zinc oxide nanoparticles were fabricated and examined in this study as a potential photocatalyst for the antibiotic ciprofloxacin (CIPF) degradation when exposed to visible LED light. The Co-precipitation technique created Cobalt-doped zinc oxide nanoparticles that were 5, 10, and 15% Co-loaded. Different known techniques have been used to characterize the synthesized ZnO and cobalt-doped ZnO nanoparticles. Compared to ZnO and other Cobalt-doped ZnO nanoparticles, the experiments showed that 10% Cobalt-doped ZnO nanoparticles were a very effective catalyst for CIPF photodegradation. According to XRD, these NPs have a hexagonal Wurtzite structure with an average size of between 38.47 and 48.06 nm. Tauc plot displayed that the optical energy band-gap of ZnO NPs (3.21) slowly declines with Co doping (2.75 eV). The enhanced photocatalytic activity of Cobalt-doped ZnO nanoparticles, which avoids electron-hole recombination, is brought on by the implantation of Co. Within 90 min, a 30 mg/L solution of ciprofloxacin was destroyed (> 99%). The kinetics studies demonstrated that the first-order model, with *R*^2^ = 0.9703, is appropriate for illuminating the pace of reaction and quantity of CIPF elimination. The recycled Cobalt-doped zinc oxide nanoparticles enhanced photocatalytic performance toward CIPF for 3 cycles with the same efficiency. Furthermore, optimization of the 10% Cobalt-doped zinc oxide nanoparticles using a Central composite design (CCD) was also studied. The optimal parameters of pH 6.486, 134.39 rpm shaking speed, 54.071 mg catalyst dose, and 31.04 ppm CIPF initial concentration resulted in the highest CIPF degradation efficiency (93.99%). Artificial neural networks (ANN) were used to simulate the experimental data. The backpropagation technique was used to train the networks with 152 input-output patterns. After experimenting with various configurations, the best results with a correlation value (*R*^2^) of 0.9780 for data validation were obtained using a three-hidden layered network that included five, five, and eight neurons, respectively.

**Supplementary Information:**

The online version contains supplementary material available at 10.1038/s41598-024-84568-w.

## Introduction

Pharmaceutical chemical residues in the aquatic environment seriously pollute the environment and can potentially cause resistance in environmental bacteria, which could then spread to pathogens, make disease treatment difficult, and pose a threat to public health^1,2^. As a result, they are a significant source of environmental pollution. They are found in groundwater, surface soil, river sediments, and wastewater after being discharged into the environment via residential, hospital, and industrial discharges such as fluoroquinolones, which are well-known for treating conditions like anthrax, digestive disorders, and tuberculosis^1^. They are only partially broken down in the body and are frequently expelled into the environment. The most popular class of quinolone antibacterial is the fluoroquinolones, which include the drug ciprofloxacin (CIPF), which is colorless^1–3^. Humans frequently receive the antibiotic CIPF, although other mammals also receive it frequently and use it for therapy. The fundamental reason for CIPF’s widespread prescription is that it may cure various infections, including skin, joints, intra-abdominal spaces, typhoid fever, and respiratory diseases^4^. Surface water flowing through pharmaceutical businesses has been discovered to contain CIPF at a high quantity (50 mg L^−1^) by the European Union Water Framework Directive (EU WFD)^5,6^. Hospital wastewater also contains pharmaceutical concentrations that are 2 to 150 times greater than those seen in municipal wastewater^5–7^.

Numerous techniques may remove certain antibiotics from water bodies, such as adsorption, nano-filtration, coagulation, electrolysis, and biodegradation^8^. However, the greatest barriers to their broad use are high operational costs and ineffective removal efficiency. Antibiotics can be broken down into tiny, harmless, and biodegradable molecules by the use of advanced oxidation processes (AOPs), which reduce their negative impact on organisms and the environment^8,9^. Additionally, hydroxyl radicals (^.^OH), when chloride (Cl), ozone (O_3_), and superoxide radicals were produced during photocatalytic degradation, AOPs have been used as efficient oxidizing agents to remove chemical compounds, notably antibiotics^8–12^. The most effective way to remove organic contaminants from water without creating hazardous intermediates is by photocatalysis. In this technique, two simultaneous processes (oxidation and reduction) lead to a photochemical reaction on the surface of metal oxide semiconductors. Several semiconductor metal oxides, including TiO_2_, ZnO, Fe_2_O_3_, WO_3_, and V_2_O_5_, have been employed as photocatalysts to remediate organic contaminants^7–12^. Compared to other semiconductor photocatalysts, ZnO and TiO_2_ are efficient for oxidizing organic pollutants. They have good chemical and photosensitivity qualities, are inexpensive, and are non-toxic^9,13^.

Due to their unique characteristics, including size, structure, and morphology, nanoparticles (NPs) have the potential to dramatically impact every area of human living^5–8^. Metal oxide nanoparticles, including iron, zinc, and cerium oxide, are just a few of the numerous varieties of these materials that are often used in medical, environmental, and pharmaceutical applications^14,15^. It has been established that, even for compounds with relatively low toxicity, the toxicity of NPs is typically higher than that of bigger particles made of the same materials^16^.

Zinc oxide has undergone considerable research as a photocatalyst due to its low cost, nontoxicity, and wide band gap, which makes it known as a multifunctional material^17^. Additionally, the Zn and O atoms in ZnO NPs have different electronegativities, further enhancing the bond’s ionicity. This can be done by doping “nitrogen” or by implanting transition metal ions^18^. Because of its high solubility in the ZnO matrix and near ionic radius (0.58) to that of Zn (0.60), cobalt (Co) is thought to be the most suitable material for doping when compared to transition metals dopants discovered thus far^18,19^. Due to Co^2+^'s maximal dipole moment value of 3B per Co ion and its five spins in the partially filled 3D shell, doping with Co^2+^ raises the magnetization levels^18^. As a result of the substitution of Co^2+^ at Zn^2+^ sites, more carriers are produced, which increases electrical conductivity when Co is added to ZnO NPs.

Adding cobalt to ZnO NPs and cobalt-doped ZnO nanoparticles (Co-ZnO NPs) controls the adsorption of molecules in aqueous solutions. It increases the material’s capacity for adsorption, where the development of active adsorption sites is facilitated by replacing the Zn^2+^ cation with the donor Co^2+^^20^. Several investigations have demonstrated cobalt dopants to decrease ZnO NP size and improve doping element solubility on the NP surface^21^. As a result, adding cobalt to ZnO NPs causes a reduction in crystallite size and may include elements with possible antibacterial properties^22^. Numerous methods, including sol-gel^23^, co-precipitation^24^, hydrothermal^25^, solvothermal^26^, combustion^27^, solid-state^28^, and numerous others, can be used to create ZnO NPs. Among them, co-precipitation has proven to be a straightforward, affordable, and quick process that is simple to adapt for industrial uses. Therefore, producing very pure nanomaterials without using risky chemical solvents, high pressures, or high temperatures is safe for the environment^29^.

Goswami et al.^13^ tested how efficiently the photocatalytic activity of the generated materials destroys the Methylene Blue (MB) dye when exposed to visible light by fabricating pure ZnO and Co-ZnO NPs with different dopant concentrations using the chemical precipitation model. They discovered that the maximal dye degradation under visible light irradiation was 98% in 180 min with 8 mol% Co-ZnO as a catalyst. When Sutanto et al.^30^ synthesized ZnO with 4 mol% of Co, they found it had the maximum photocatalytic activity, degrading methylene blue (MB) by nearly 76.31% after two hours while exposed to UV light irradiation. The Co-precipitation approach was used by Nair et al. to produce pure and cobalt doping ZnO NPs, and they investigated the decomposition of methylene blue under the UV region^16^. ZnO and Co-ZnO NPs were prepared by the Co-precipitation method with various concentrations of cobalt and were studied its structural, photocatalyst, optical, and antibacterial properties and its application for decomposition of methyl green dye under UV light^31^. Our batch study demonstrated that Co-ZnO is particularly effective at degrading the antibiotic CIPF. It is still necessary to do further scientific research on the variables that affect CIPF reduction.

Structure is another factor that categorizes antibiotics and antimicrobials, influencing how easily they degrade in the environment^22^. Similarly, 90% of the most often used antibiotic classes are represented by amoxicillin, azithromycin, cefixime, and ciprofloxacin, so their environmental leakage has the most significant impact^29^. In the current work, one of these newly developed antibiotics, CIPF, is employed for photocatalytic degradation utilizing a Co-ZnO photocatalyst under visible light. For the CIPF antibiotic, several factors were optimized, including the dosage of the Co-ZnO photocatalyst, the pH of the reaction matrix, the starting antibiotic concentration, and the photocatalyst dose. Comprehensive studies that concentrate on applying Co-doped ZnO as a visible light-responsive photocatalyst for CIPF degradation take time to come by. Most research in this field often focusses on different types of photocatalysts, such as bare ZnO or TiO_2_. Eventually, additional investigation is required to find the ideal cobalt doping concentrations in ZnO to maximize photocatalytic activity in the presence of visible light. This paper investigated the effect of various doping concentrations on the degrading efficiency of CIPF.

To assess the efficacy of this tactic. Building experiments to determine the impact of various elements and get the optimal circumstances in a restricted number of planned tests is done using response surface methodology (RSM), founded on statistical and mathematical approaches. The most popular method in the RSM technique is central composite design (CCD). Many improvements to the pollutant removal process have been observed using the RSM to optimize critical parameters^32^.

Artificial Neural Network (ANN) modeling is statistical software inspired by the biological neural networks in human brains. ANN modeling predicts complicated patterns and correlations for huge input and output datasets. Various ANN models exist, but the feed-forward back-propagation NN (BPNN) is the most common. The BPNN’s main advantage is facing the over-fitting and error correction of the ANN algorithm in applications. The BPNN model is composed of an input layer (IL) (independent variable), hidden layers (HNs), an output layer (OL) (dependent variable), the connection weight and biases, the activation function, and the summation nod. The nodes are the main components of the input, hidden, and output layers. These nodes are known as neurons and are utilized for storing and processing information. The neurons transfer the input signals through the activation function to get the output signals. One of the most critical components of the ANN model is the hidden layer, thanks to its significant impact on the ANN model efficiency in predicting the statistical relation between input and output variables. The hidden layer inhibits the non-linearity between input and output variables. Two dominant factors are used to determine the efficiency of the ANN model the correlation coefficient (*R*^2^) and mean square error (MSE). The highest *R*^2^ and lowest MSE are the main properties of the best-fit ANN model^33,34^.

The current paper presents evidence of the photocatalytic activity of cobalt-doped zinc oxide nanoparticles synthesized by co-precipitation method, in the photodegradation of CIPF in water under LED light. It does this by examining the structural, optical, and photocatalytic properties of ZnO nanoparticles (5, 10, and 15%) doped with cobalt and ZnO NPs. Furthermore, the RSM-CCD model was utilized to optimize the removal of CIPF from water by photodegradation processes when exposed to visible light. To the best of the authors’ knowledge, this is the first study to optimize the photocatalytic degradation of CIPF utilizing chemically synthesized Co-doped ZnO and RSM-CCD.

## Materials and methods

### Chemicals and equipment

The following materials were acquired from Merck, USA: zinc acetate dihydrate (99.999%), cobalt acetate tetrahydrate (98%), NaOH, isopropanol (IPA) (99.9%), sodium-ethylenediaminetetraacetic acid (Na-EDTA), and benzoquinone (BQ). Amirya Pharmaceuticals, Egypt, provided the CIPF 200 mg/100 mL IV infusion solution that was bought. The ZnO and Co-ZnO NP photocatalyst samples were identified using the following equipment. The XRD Bruker Meas Srv, Germany (D2-diffractometer, 2^nd^ Gen, that controls at 30 kV, 10 mA using Cu tube *λ* = 1.5418 Å and 2*θ* with a temperature range of 5 to 80°) was used to validate the crystallinity and average crystal size of ZnO and Co-ZnO NPs. Bruker VERTEX70 connected to Platinum ATR V-100 model, Germany, was used for Fourier transform infrared (FTIR) spectroscopy sample analysis in the 400–4000 cm^−1^ wavenumber range. The shape and surface properties of the materials were assessed using scanning electron microscopy (SEM) (SEM-JEOL, IT 200, Japan) fitted with energy dispersive X-ray spectroscopy (EDX) for elemental analysis. Utilizing Transmission Electron Microscopy (TEM) (JTM 1400 plus-Japan), the dimensions and forms of the nanostructures were ascertained. Using a GBC Cintra 3030 spectrophotometer working in the UV-visible range between 190 and 900 nm, the optical absorbance of these materials was determined. The mean pore width and specific surface area (Brunauer Emmett-Teller, or BET) were calculated using the BELSORP - Mini II from BEL Japan, Inc. The materials were subjected to thermal studies using the SDT650-Simultaneous Thermal Analyser apparatus, which ramped up the temperature by 10 °C per minute. X-ray photoelectron spectroscopy (XPS) was recorded on K-ALPHA (Thermo Fisher Scientific, USA) with monochromatic X-ray Al K-alpha radiation of 10 to 1350 eV, spot size 400 micro m, pressure 10^−9^ mbar, whole spectrum passes energy 200 eV, and at narrow spectrum 50 eV.

### Formation of ZnO and Co-doped ZnO (Zn_1-***x***_Co_***x***_O)

After adding Solution-A (0.5M Zn (CH_3_CO_2_)_2_·2H_2_O) to distilled water (DW) (100 mL) and stirring at 60 °C for 30 min, 0.5M NaOH was added dropwise until a white color precipitate was evident. At the same temperature, the solution was continuously stirred for 5 h. The white precipitate was separated, filtered, washed with DW and EtOH, and allowed to dry at 100 °C for 24 h. ZnO NPs were produced by calcining the obtained powder at 500 °C for 3 h^10,35,36^. The ZnO NPs pale white powder was meticulously assembled and stored till use (3.87 g, 95%). The cobalt doping ZnO (Zn1-xCoxO) NPs were also produced using the co-precipitation approach. The appropriate quantity of Co(CH_3_CO_2_)_2_^.^4H_2_O (Solution-B) was dissolved in distilled water while being stirred for 30 min to create various ratios (5, 10, 15% of Co/ZnO). After that, solution B (sample-wise) was added to solution A (zinc acetate), and the identical methods for the synthesis of ZnO NPs were followed, beginning with precipitation and concluding with three hours of calcination at 500 °C^10,14,16,37^.

### Photocatalytic activity

To ascertain the optimal catalyst performance, a 300 mL Pyrex glass conical containing CIPF (100 mL) of 30 mg/L concentrations at neutral pH was filled with a specific amount of ZnO and 5, 10, and 15% of Co-ZnO NPs. The beaker was then exposed to a LED light source for two hours under (150 W LED-light lamp) as a visible LED-light source and the removal efficiency of CIPF was measured. The photocatalyst and CIPF solution were combined in a conical flask (100 mL) for the photocatalytic degradation experiment. The mixture was then exposed to a dark environment for 30 min to establish the equilibrium between adsorption and desorption. Then, 2 mL of the antibiotic solution was added to a mixture containing the photocatalyst at predefined intervals to expose it to visible light. The concentration of CIPF at a wavelength of *λ*_max_ 270 nm was measured using a Pg/T80 UV-Vis spectrophotometer model, following a 30-min centrifugation period at 6000 rpm.

1 $$Degradation\;efficiency = \frac{{C_{0} - C_{t} }}{{C_{0} }} \times 100$$

where *C*_0_ represents the CIPF initial concentration in water, and *C*_t_ indicates the CIPF concentration at the specified time intervals after irradiation^13^. By adjusting the pH, catalyst doses, temperature, shaking speed, and antibiotic concentrations during photodegradation, researchers were able to define the ideal settings for effective photocatalytic degradation.

### Radical scavenger

Three scavengers were individually added to a 100 mL, 30 mg/L CIPF solution in order to quench photo-generated species (holes, h^+^), hydroxyl radicals (^•^OH), and superoxide radicals (^•^O^2−^), each of which is responsible for catalytic degradation^11^.

### Experimental design

Four factors in this work are considered as important variables that influence the photocatalysis efficiency. The central composition design (CCD) was employed by the design expert program (version 13.0.5.0) to optimize the independent variables, which included the catalyst dosage, beginning antibiotic concentration, pH level, and shaking rate. Equation (2) was used to calculate how many experiments would be needed for four parameters^38^:

$$\begin{aligned} {2}^{{\text{n}}} + {2}^{{{\text{n}} + {6}}} & = {2}^{{4}} \left( {{16}\;{\text{tests}}\;{\text{for}}\;{\text{factorial}}\;{\text{points}}} \right) + {4} \times {2}\left( {{8}\;{\text{tests}}\;{\text{for}}\;{\text{axial}}\;{\text{points}}} \right) \\ & \quad + {6}\left( {{\text{central}}\;{\text{points}}} \right) = {3}0,n = {4}. \\ \end{aligned}$$

A quadratic polynomial equation was used to compute the four parameters listed in Eq. (2) using CCD^38,39^.

2 $$\begin{aligned} {\text{Y}}\left( \% \right) & = {\text{b}}_{0} + {\text{b}}_{{1}} {\text{A}} + {\text{b}}_{{2}} {\text{B}} + {\text{b}}_{{3}} {\text{C}} + {\text{b}}_{{4}} {\text{D}} + {\text{b}}_{{{12}}} {\text{AB}} + {\text{b}}_{{{13}}} {\text{AC}} + {\text{b}}_{{{14}}} {\text{AD}} + {\text{b}}_{{{23}}} {\text{BC}} + {\text{b}}_{{{24}}} {\text{BD}} + {\text{b}}_{{{34}}} {\text{CD}} \\ & \quad + {\text{b}}_{{{11}}} {\text{A}}^{{2}} + {\text{b}}_{{{22}}} {\text{B}}^{{2}} + {\text{b}}_{{{33}}} {\text{C}}^{{2}} + {\text{b}}_{{{44}}} {\text{D}}^{{2}} \\ \end{aligned}$$

The intercept is shown by b_0_, and Y represents the variable response. In addition to b_11_, b_22_, b_33_, and b_44_ being quadratic coefficients, b_1_, b_2_, b_3_, and b_4_ are independent variable coefficients. The coefficients for interactions are b_12_, b_23_, b_13_, b_14_, b_24_, and b_34_. Furthermore, the amounts of the catalyst, the antibiotics, the rate of shaking, and the pH level are the independent factors, whereas A, B, C, and D are the dependent variables (Table 1).

**Table 1:** Independent variable values at low and high levels.

Factor	Name	Units	Minimum	Maximum	Mean	Std. Dev. +
A	10% Co-ZnO NPs dosage	mg	20	100	60	18.19
B	CIPF dosage	mg/L	10	50	30	9.10
C	Shaking speed	rpm	50	250	150	45.49
D	pH		3	11	7	1.82

### ANN modelling

**Fig. 1:**
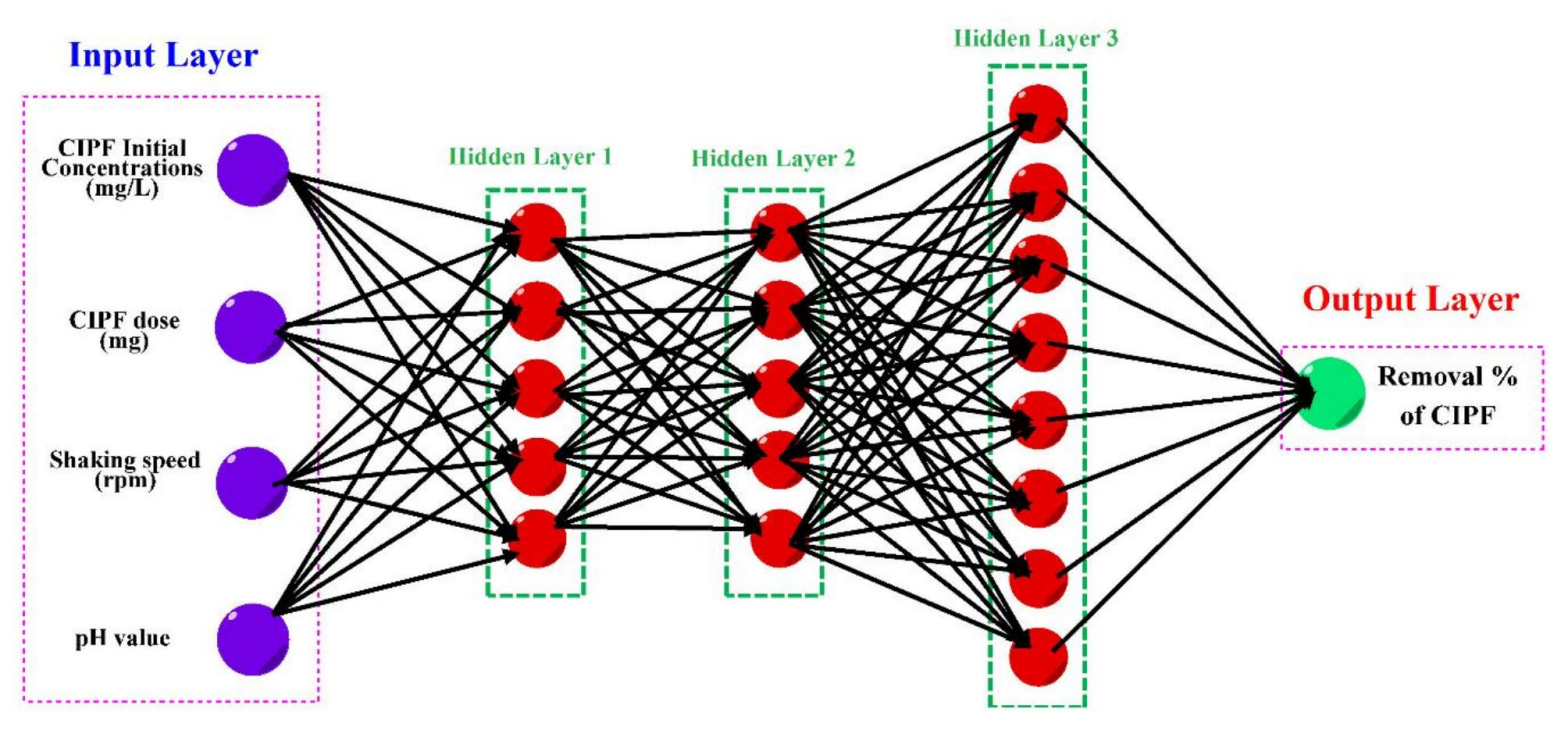
ANN net was utilized in this study.

This paper used the ANN model to study the photodegradation of antibiotic ciprofloxacin by exposing it to visible light and using fabricated cobalt-doped zinc oxide nanoparticles as a catalyst. ANN computations were calculated using MATLAB R2015b version. The ANN model is trained by the Levenberg Marquart (LM) training algorithm with training (70%), testing (15%), and validation (15%). The best-fit ANN model architecture was the BPNN, which has three hidden layers. The first, second, and third hidden layers had 5, 5, and 8 neurons, respectively (Fig. 1). This ANN model showed the best performance thanks to the highest *R*^2^ and the lowest MSE values. A vast number of hidden neurons (HL) were tested. The number of tested hidden neurons was 4–24 hidden neurons. Finally, the Catalyst dosage of the fabricated cobalt-doped zinc oxide nanoparticles (mg), shaking speed (min), pH, and antibiotic dosage of ciprofloxacin (mg) were the independent variables. The removal of antibiotic ciprofloxacin was the dependent variable^33,34^.

## Result and discussion

### FTIR analysis

**Fig. 2:**
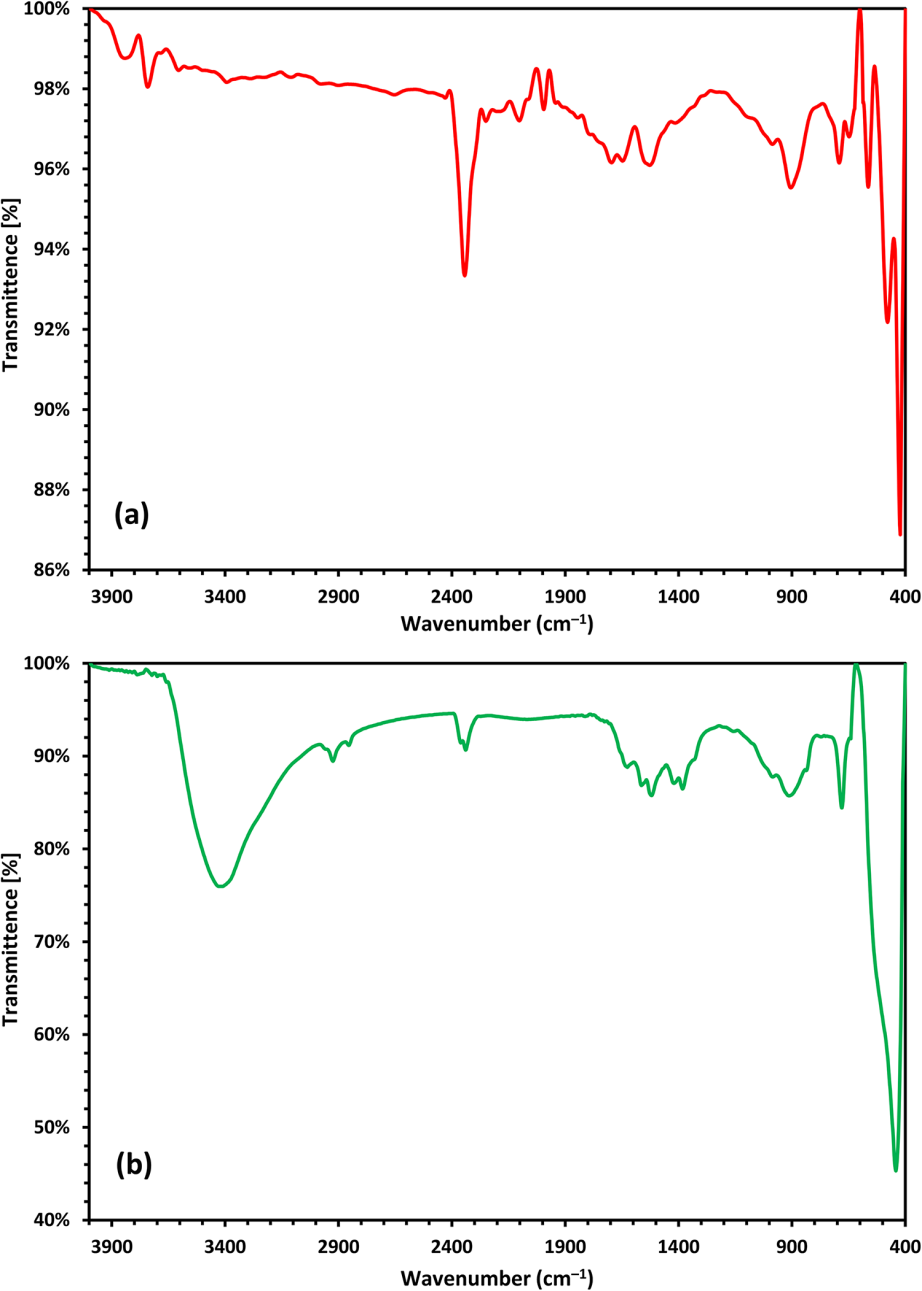
FTIR analyses of (**a**) ZnO NPs and (**b**) Co-ZnO NPs catalyst.

The ZnO NPs and 10% Co-ZnO NPs catalysts FTIR analyses are existing in Fig. 2a, b. Nearly all samples have comparable FTIR spectra, which reveal the existence of the same functional groups. On the other hand, when the dopants concentration increases, the fraction of some functional groups increases^38^. The Zn-O stretching band for ZnO NPs can be attributed to the peaks at 422.9, 478.32, and 563.44 cm^−1^, which confirm that the ZnO NPs are positively generated^40^. The bands at 3842.72 and 3741.36 cm^−1^ indicate water has been adsorbed on the surface of the NPs and correspond to O-H stretching of the OH group^40,41^, but the band at 1695 cm^−1^ displays the existence of the O-H bending vibration of H_2_O molecules (adsorbed)^42^. CO_2_ in the air can be attributed to the band between 2343.37 and 2250.01 cm^−1^. C-O stretching is responsible for the absorbance peak at 1527 cm^−1^^41^. For 10% Co-ZnO NPs, the O-H stretching mode of water molecules in the Co-O-Zn lattice is responsible for the absorption peak that emerged at 3424.19 cm^−1^. The presence of moisture (H_2_O molecules) may be what causes this band to exist. A minor quantity of water on the surface of Co-ZnO NPs is responsible for the H-O-H bending vibration responsible for the band seen at 1628.48 cm^−1^. The band shown below at 440.78 cm^−1^ may be caused by the Zn-Co-O lattice’s stretching mode^43^. The band at 680.64 cm^−1^ that depicts Co-O stretching in both doped samples verifies the existence of Co. The findings support past research and show NPs form naturally without contamination^41^.

### BET analysis

The BET surface area^15,17,36^ is used to explore the porous nature of the ZnO-NPs, 5, 10, and 15% Co-ZnO NPs samples that have surface areas of 7.325, 37.876, 32.033, and 32.855 m_2_/g, respectively, and monolayer volumes (*V*_m_) of 1.683, 8.702, and 7.549 cm^3^ (STP) g^-1^. The mean pore diameters are 11.05, 16.28, 13.82, and 18.38 nm, and the volume of total pores is 2.023 10–2, 15.42 10–2, 11.06 10–2, and 15.10 10–2 cm^3^/g, respectively (Table S1, Fig. S1). The mesoporous material’s characteristics are align with pores with an average diameter of 50 nm (Table 2).

**Table 2:** BET and BJH analyses of ZnO and 10% Co-ZnO NPs samples.

Model	Parameter	ZnO NPs		10% Co-ZnO NPs	
BET	*a*_s, BET_ (m^2^∕g)	7.325		32.033	
	*V*_m_ (cm^3^ STP)/g)	1.683		7.360	
	Mean pore diameter *P*_m_ (nm)	11.051		13.817	
	Volume of total pore *V*_T_ (cm^3^/g)	2.023 × 10^−2^		11.06 × 10^−2^	
BJH	*V*_p_ (cm^3^/g)	2.048 × 10^−2^		11.12 × 10^−2^	
	*a*_p_ (m^2^/g)	7.910		34.278	

### Scanning electron microscope (SEM)

**Fig. 3:**
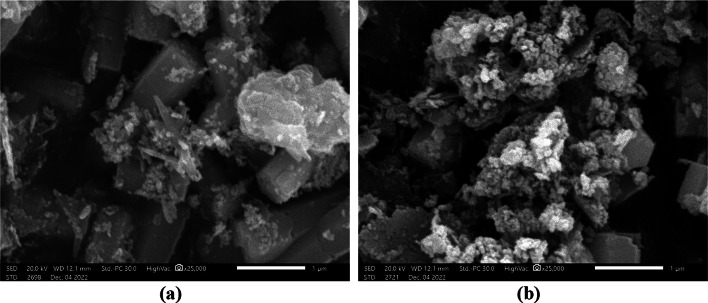
SEM pictures of (**a**) ZnO NPs and (**b**) 10% Co-ZnO NPs at high vacuum (20.0 kV, 25,000 magnification).

Using SEM imaging, the external morphology of each nanostructure was investigated. Figure 3 displays the morphology of SEM images of synthesized 10% Co-ZnO and ZnO NPs. The SEM images of ZnO NPs in Fig. 3a show a cuboid shape. SEM scans in Fig. 3b depict how the surface morphology of 10% Co-ZnO NPs has changed by adding cobalt. ZnO sample microstructures are likely to change because of Co^2+^ ion doping; however, this change typically leads to nanoparticle agglomeration. An increase in the attractive force between the NPs and a rise in the surface area to volume ratio appeared to cause the particles to group^41^, which causes particles to appear to cluster together. Consequently, tiny cobalt particles formed on top of larger clusters.

### EDX analysis

**Fig. 4:**
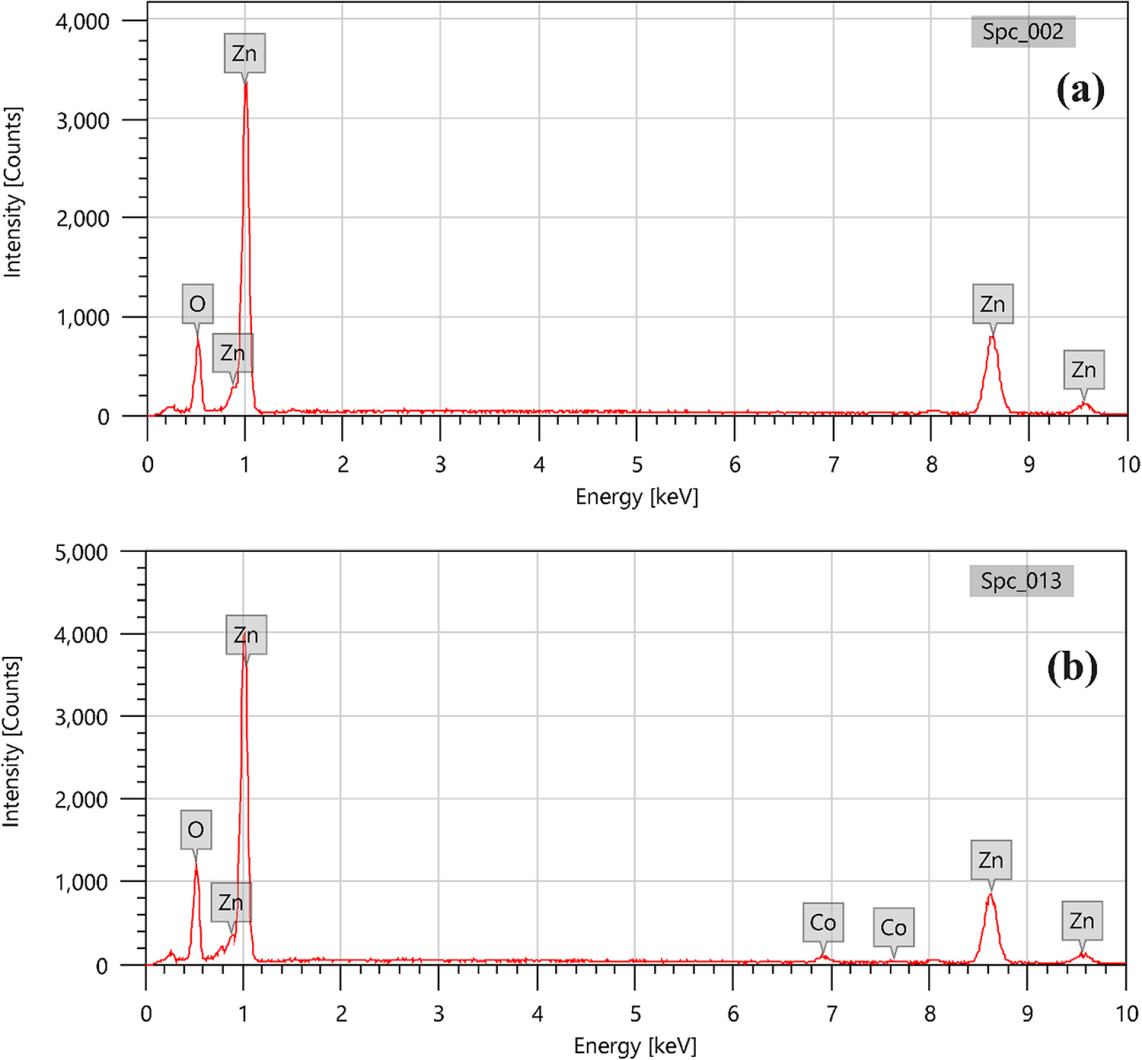
EDX analyses of (**a**) ZnO NPs and (**b**) 10% Co-ZnO NPs using Landing Voltage 20.0 kV, WD 10.0 mm, and Magnification × 5,000 under High Vacuum.

The chemical composition and manufacture of ZnO and 10% Co-ZnO NPs were examined using EDX. Figure 4 displays typical EDX spectra of ZnO and Co-ZnO NPs. The formation of un-doped ZnO NPs was demonstrated by the EDX spectra, which also demonstrated the existence of Zn and O chemical constituents in the ZnO NPs, demonstrating the extreme purity of the synthesized samples. Quantitative EDX results (Table 3), show the weight percent of 82.75 ± 0.98 and 17.25 ± 0.25 for Zn and O elements. For 10% Co-Zno NPs, the peaks appear at 74.49 ± 0.86, 22.50 ± 0.25, and 3.01 ± 0.13 for ZnO and Co. The strong peaks of EDX showed that the synthesized NPs had crystalline structures, and the EDX peak placements were compatible with ZnO. The strong intensity and small breadth of ZnO diffraction peaks indicate that the final products were extremely crystalline.

**Table 3:** ZnO NP and 10% Co-ZnO NP Element analysis utilizing EDX analysis.

Element	ZnO NPs		10% Co-ZnO NPs	
	Mass%	Atom%	Mass%	Atom%
Zinc	82.75 ± 0.98	54.00 ± 0.64	74.49 ± 0.86	43.88 ± 0.52
Oxygen	17.25 ± 0.26	46.00 ± 0.66	22.50 ± 0.25	54.15 ± 0.61
Cobalt	0.00	0.00	3.01 ± 0.13	1.97 ± 0.09
Total	100.00	100.00	100.00	100.00

### Transmission electron microscopy (TEM)

**Fig. 5:**
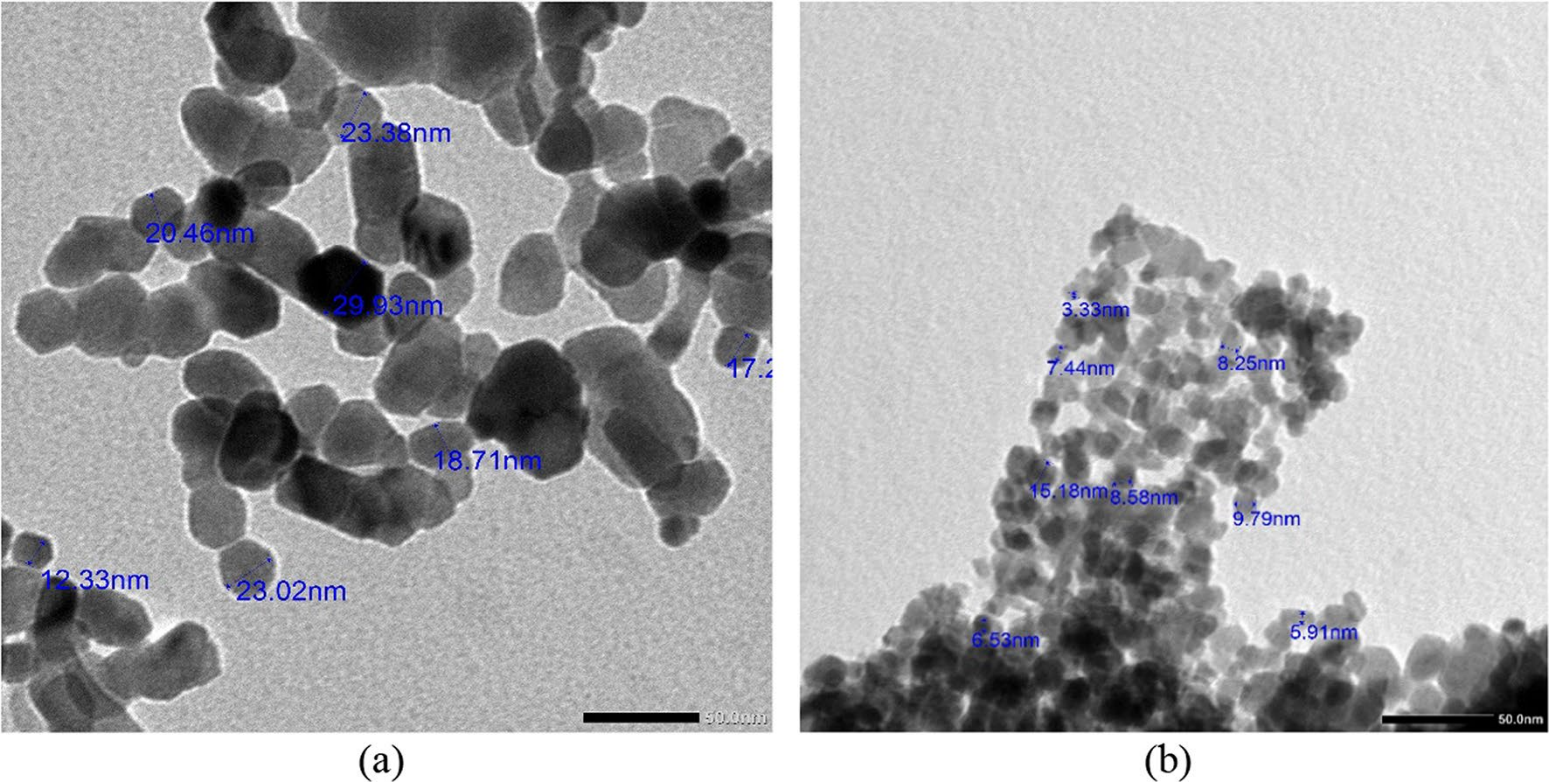
TEM images of (**a**) ZnO NPs and (**b**) 10% Co-ZnO NPs under × 100,000 magnification.

The size and shape of the synthesized ZnO and 10% Co-Zno NPs were examined using TEM. The TEM image of ZnO and 10% Co-Zno NPs with a particle size is displayed in Fig. 5. Figure 5a shows a TEM picture of variously sized ZnO NPs, including radial, triangle, rod, hexagonal, and rectangle shapes. The ZnO NPs size is shown in Fig. 5b as being between 12 and 30 nm. As cobalt concentrations increased, the average particle size decreased from 30 to 15 nm. The particle shape is deformed when co-atoms are doped into the hexagonal ZnO lattice^19^.

### X-ray diffraction (XRD) analysis

**Fig. 6:**
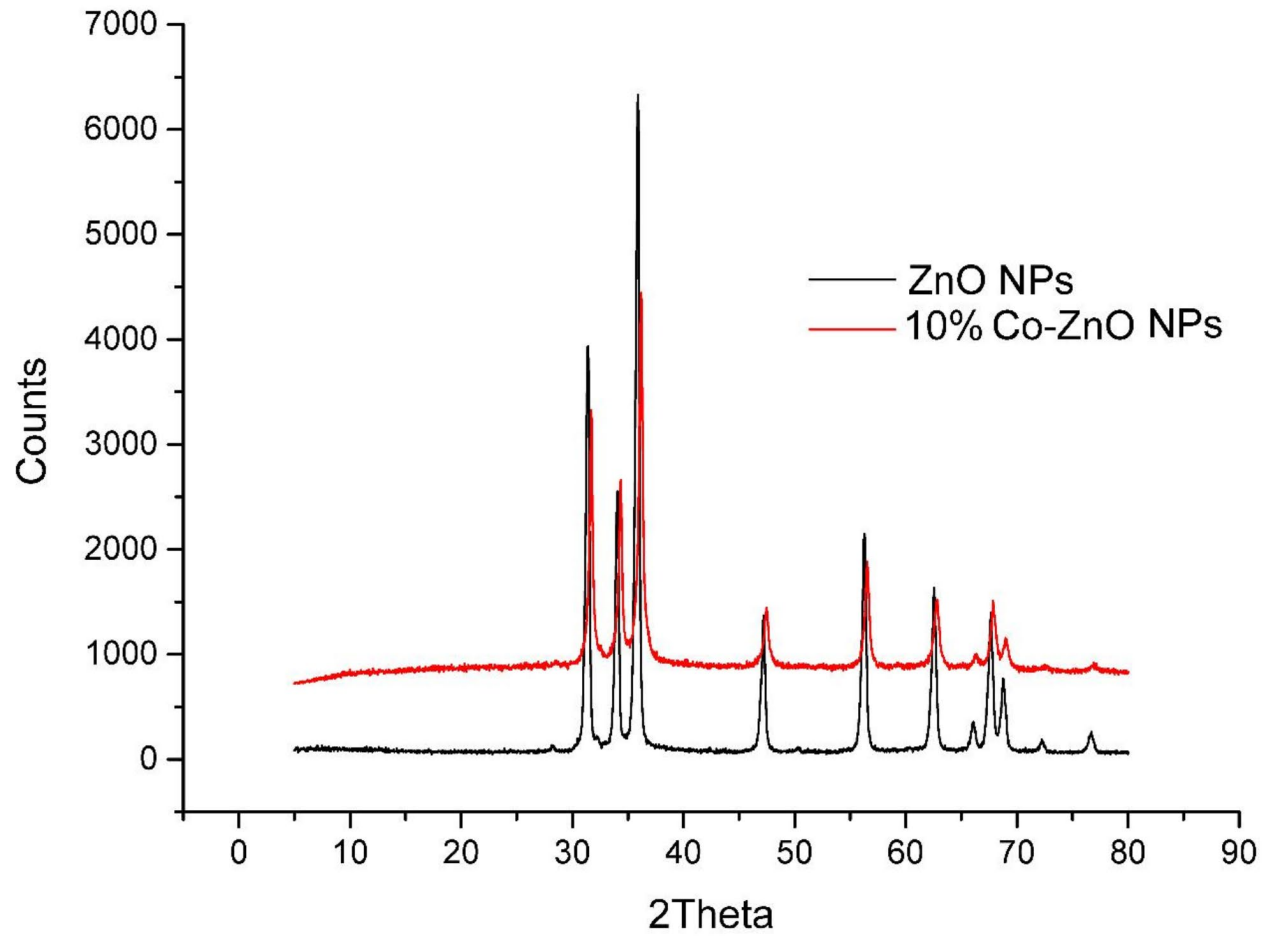
10% Co-ZnO NPs and ZnO NPs with an XRD pattern that ranges from 5 to 80° are operated at 30 kV and 10 mA using a Cu tube (*λ* = 1.54 Å).

The ZnO NPs and Co-ZnO NPs (Co = 5, 10, and 15%) size and crystal structure are determined by the phase and lattice spacing (d) of the crystalline material unit cell, which is determined by XRD analysis. An XRD pattern comprises a range of diffraction peaks with different strengths and angles that serve as a fingerprint for a particular crystalline substance. Figure S2 and Fig. 6 depict the crystalline phase of the produced NPs, indicating that all of the peaks in the hexagonal Wurtzite structure according to COD9008877 Card, are indexed to the same point and that there are no longer any diffraction peaks of unknown impurities such as Co, CoO, or Co_3_O_4_ phases. This proves that the Co^2+^ ions in the ZnO lattice have been substituted into Zn^2+^ ions without altering the ZnO’s Wurtzite structure host. Zn^2+^ ions are replaced with Co^2+^ ions because their ionic radius (0.58 A) is less than Zn^2+^ ions’ (0.60 A). The unit cell collapses as a result of the ZnO NPs’ peak intensities shifting slightly towards higher values to create place for these heterogeneous Co^2+^ ions in the crystal lattice of ZnO NPs. The prominent peaks of the crystal planes (100), (002), and (101) shift to greater diffraction angles as the concentration of Co dopant rises (Fig. 6)^16^. The average crystallite size "D" of the pure ZnO and Co-ZnO NPs samples was determined using Debye-Scherrer’s method from the highest diffraction peak (101) by Eq. (3)^13,42^.

3 $$D = \frac{K\lambda }{{\beta Cos\theta }}$$

where *λ* is the wavelength (= 1.54178 Å) of X-rays, *K* is a constant (*K* = 0.94 for spherical shape),* θ* is the Bragg’s diffraction angle, and *β* is the full-width at half-maxima (FWHM) of the diffraction peak (in radians)^13^.

The typical crystallite size increases from 38.47 to 48.06 nm when cobalt concentration rises (Table S2). In light of the aforementioned information, it is clear that as Co concentration grew, so did the average crystal size. Increasing the concentration of Co in the ZnO matrix might be the reason for this since it would encourage particle nucleation and make it easier to produce Co-ZnO NP granules. The outcomes of earlier studies are consistent with this one^22,31^.

### UV-visible diffuse reflectance spectroscopy

**Fig. 7:**
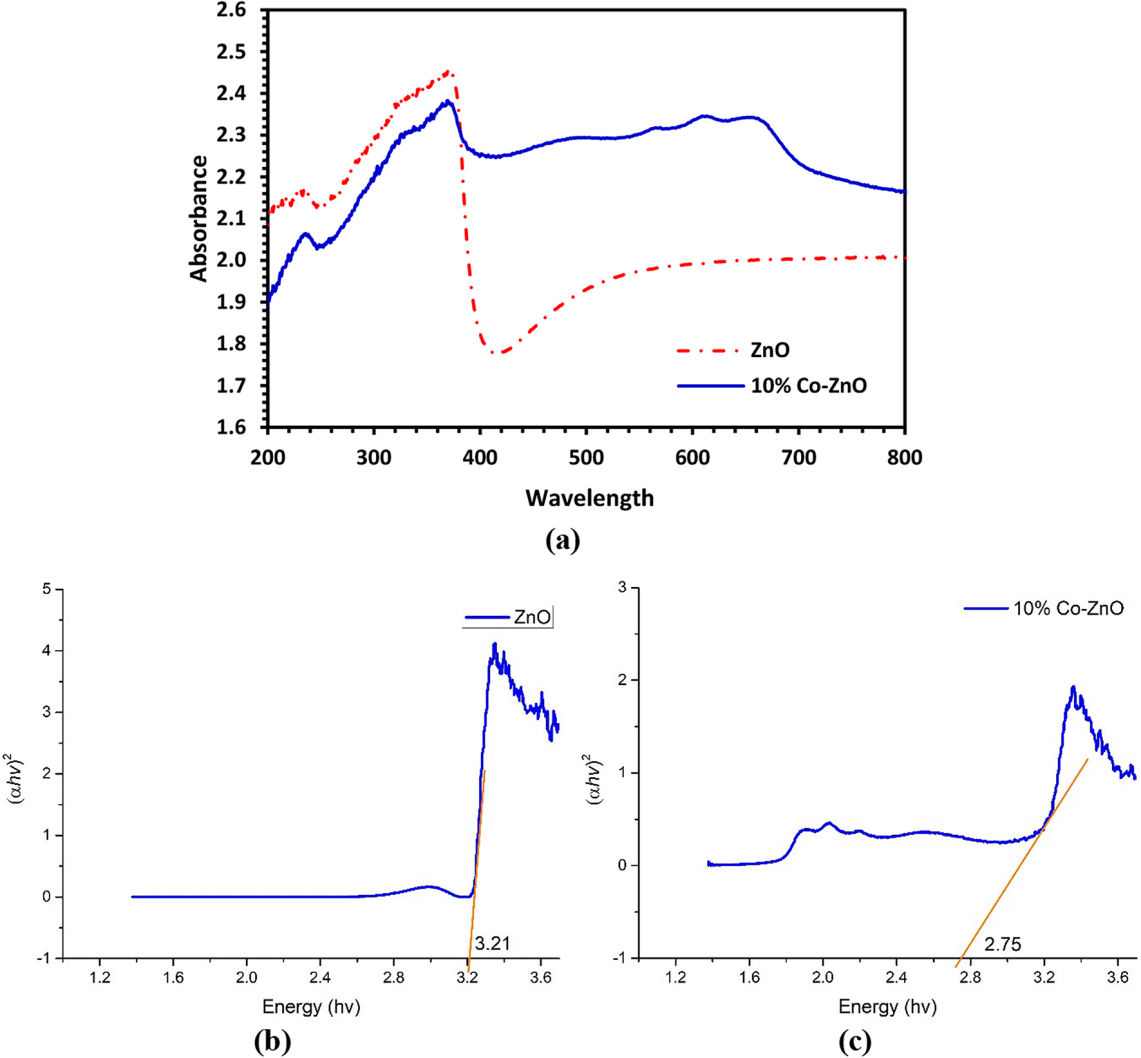
(**a**) DRS UV/vis spectrum of ZnO and 10% Co-ZnO NPs using wavelength scan from 200 to 800 nm. Tauc plots of (**b**) ZnO NPs and (**c**) 10% Co-ZnO NPs.

The UV-visible absorption spectra of 10% Co-ZnO NPs and pure NPs are shown in Fig. 7 for the wavelength range of 190 to 900 nm. These spectra were examined to ascertain the changes that Co doping has made to the energy band structure of ZnO NPs. These data mainly aim to calculate the band gaps of various materials (*E*).The spectrum of ZnO NPs exhibits the typical strong ZnO band gap absorption edge at about 371 nm (3.3 eV). Co-ZnO NPs exhibit three transition peaks at 566, 613, and 655 nm, which are attributed to the ^4^A_2_(F) → ^2^A_1_(G), ^4^A_2_(F) → ^4^T_1_(P), and ^4^A_2_(F) → ^2^E(G) d-d crystal field transitions (Fig. 7)^18^. These changes originate from the shift of the Co^2+^ ions to the d-d (3d7) electronic state from the tetrahedral coordinated high-spin state (S = 3/2), which can be observed in Fig. 7, the Co^2+^ absorption band’s general position (550–700 nm) likewise increases when Co loading increases. The transitions seen in the absorption spectra of the NPs indicated that Co2 + ions had efficiently replaced the Zn^2+^ ions in the hexagonal ZnO Wurtzite. The absorption spectra’s redshift revealed that when the concentration of Co increased, the *E* value decreased (Eq. 4)^18^.

4 $$E = \frac{hC}{\lambda\!\!\!^-}$$

where *C* is speed of light (2.998 × 108 ms^−1^), *h* is the Planck constant (6.626 × 10^−34^ J s), and λ is the cut-off wavelength in nm. The results of this investigation are strikingly comparable to those of studies conducted by Naik et al.^18^, Manandan et al.^31^, and Sutanto et al.^30^, which found that lowering the energy band-gap (*E*) value is caused by increasing the concentration of Co over ZnO. Figure 7b, c shows the (*αhυ*)^2^ values concerning to photon energy (*hυ*). ZnO NPs and 10% of Co- ZnO NPs were concealed to have optical direct energy band-gaps (*Eg*) of 3.21 and 2.75 eV, respectively.

### X-ray photoelectron spectroscopy (XPS)

**Fig. 8:**
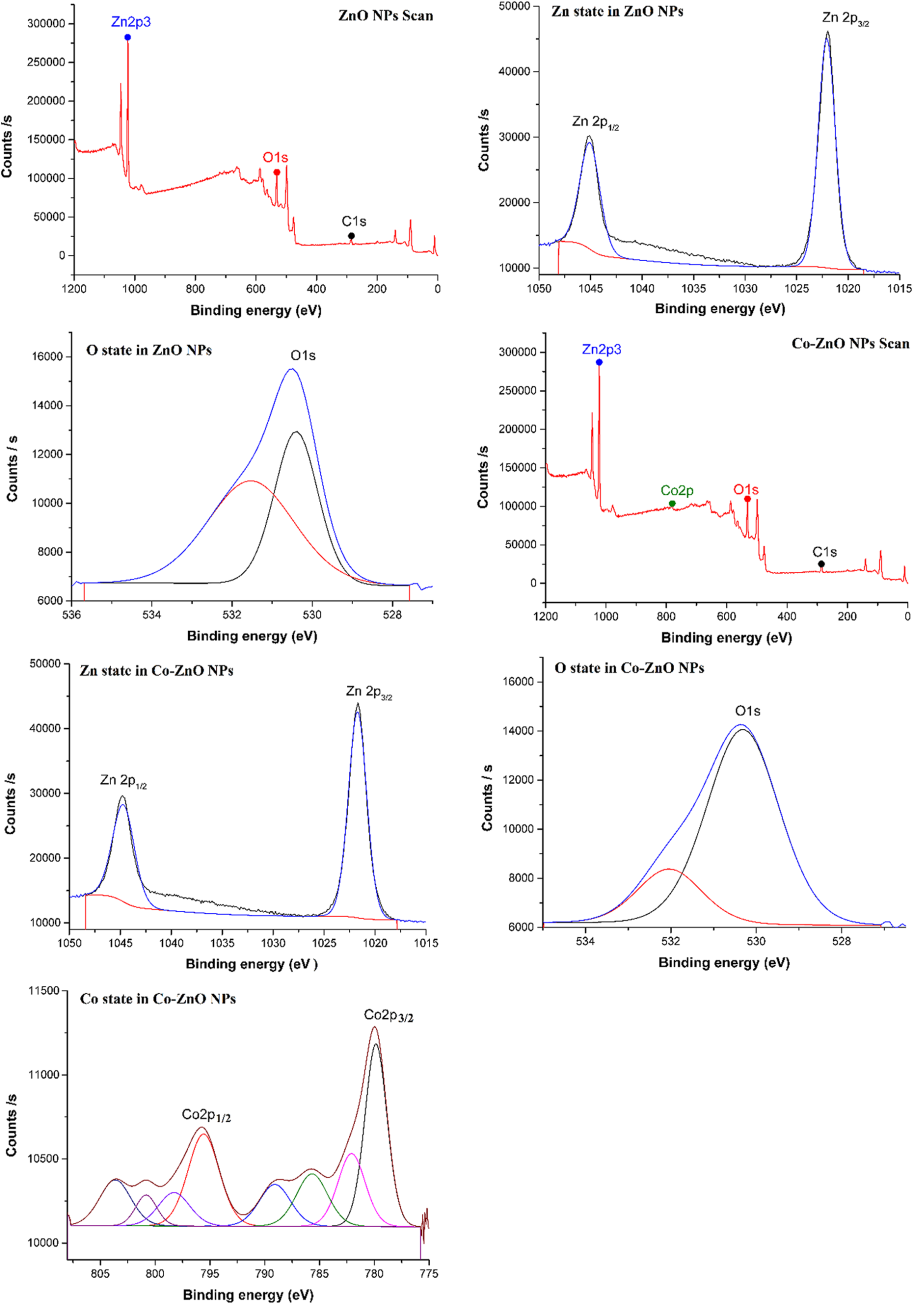
ZnO NPs and Co-ZnO NPs were subjected to XPS examination employing monochromatic X-ray Al K-alpha radiation with a narrow spectrum of 50 eV, 400 µm spot size, 10^−9^ mbar, and 200 eV energy.

To confirm that Co. existed and was in its proper oxidation state in the samples, XPS analysis of the ZnO and Co-ZnO NPs was conducted. The ZnO’s survey spectrum (Fig. 8) shows that it was made up of Zn, O, Co, and C, with the possibility that C resulted from CO_2_ adsorption on the sample surfaces. When no peaks in the XPS spectra match any extra elements, the compounds produced are pure. Two distinct peaks, with the highest peak for Zn 2p_3/2_ and the lowest peak for Zn 2p_1/2_, can be seen in the XPS spectra of the electrons in the orbital Zn 2p. For the ZnO, the binding energies for Zn 2p_3/2_ and Zn 2p_1/2_ are 1022.07 and 1045.06 eV, respectively. This finding suggests that the Zn^2+^ ion predominates on ZnO NPs’ surface. Furthermore, the binding energies of Zn for the 10% Co-ZnO NPs are 1021.72 and 1044.69 eV, respectively^43^, which correspond to Zn 2p_3/2_ and Zn 2p_1/2_. Zn is present as the Zn^2+^ state, as shown by the spin-orbital splitting of Zn 2p_3/2_ and Zn 2p_1/2_, which is found to be 23.1 eV^18,38^.

The oxygen (O1s) narrow spectrum is depicted in Fig. 8 as it passes through peaks in the binding energy ranges of 530.4 to 531.53 eV. The most noticeable band in the lower binding energy area is caused by oxygen atoms at the hexagonal ZnO’s regular lattice site in this configuration, which is 530.4 eV. Zn(OH)_2_ is believed to be responsible for a slight peak at the side of higher binding energy (531.53 eV). Moreover, as seen in Fig. 8, the XPS survey spectrum revealed Co-related peaks at 779.84 and 785.67 eV, corresponding to the Co 2p_3/2_ and Co 2p_1/2_ nodes, respectively. Additionally, there is a 5.83 eV difference between the two corresponding peaks. This finding suggests that Co can take the role of Zn in the cation position by oxidizing to Co^2+^. Because of this, the samples that were made have all been subjected to XPS examination, which reveals that all of the nanostructures are of great purity and have not produced any secondary phases^28,44–46^.

### Thermal analysis

**Fig. 9:**
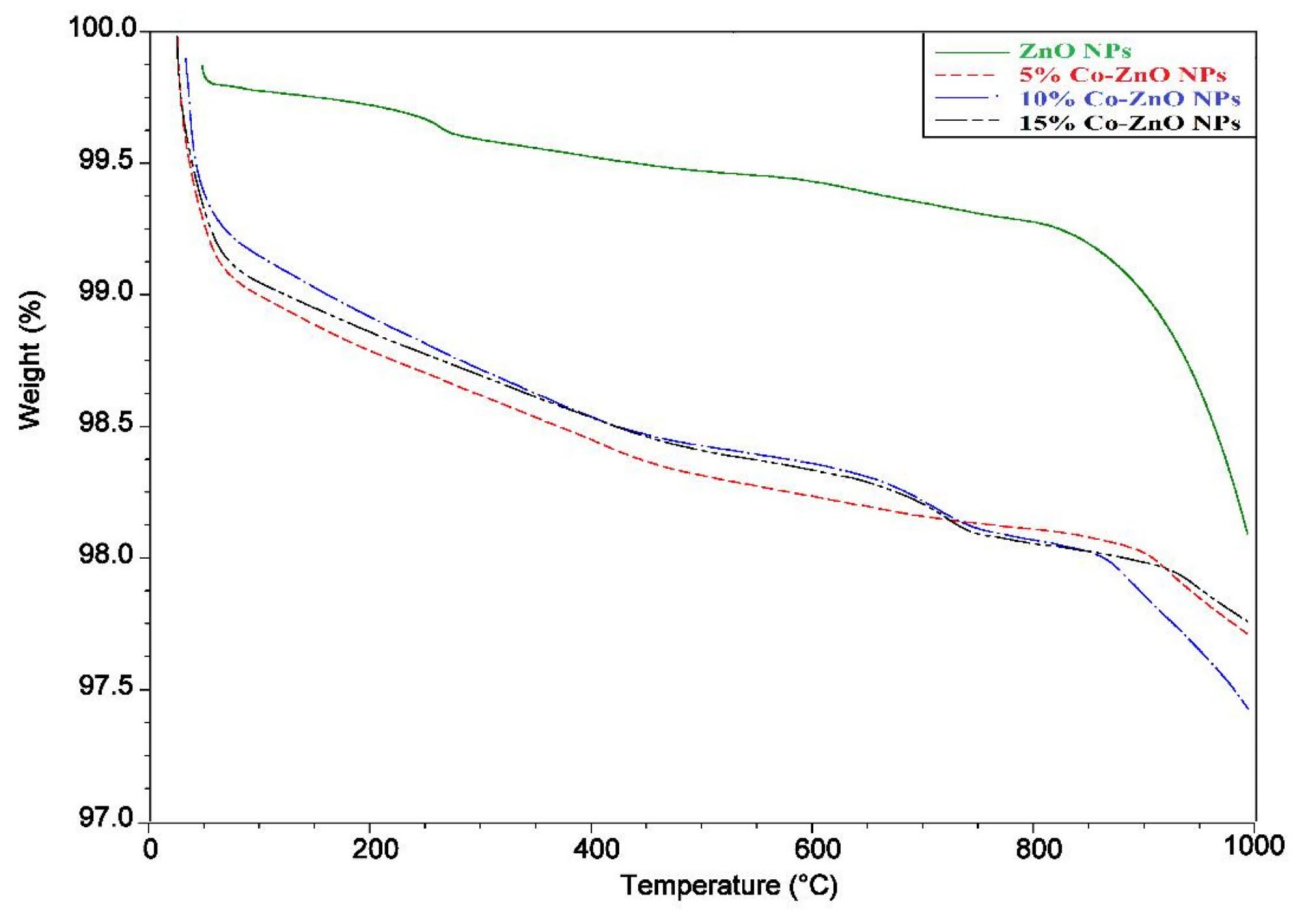
ZnO NPs and 5, 10, and 15% Co-ZnO NPs samples TGA analysis from room temperature to 1000 °C using 10 °C ramping temperature.

The biomass thermal stability of samples (ZnO NPs and Co-ZnO NPs) was examined using TGA, which is a commonly used method for determining biomass thermal deterioration (Fig. 9). The first weight loss from samples is due to moisture loss between 50–250 °C^47^, and the remaining weight loss occurs between 250 and 1000 °C, the resulting loss of 1.498 and 2.471% of its weight, respectively (Fig. S3). After losing weight, the samples have high stability.

## Photocatalytic studies

### Photocatalytic test

**Fig. 10:**
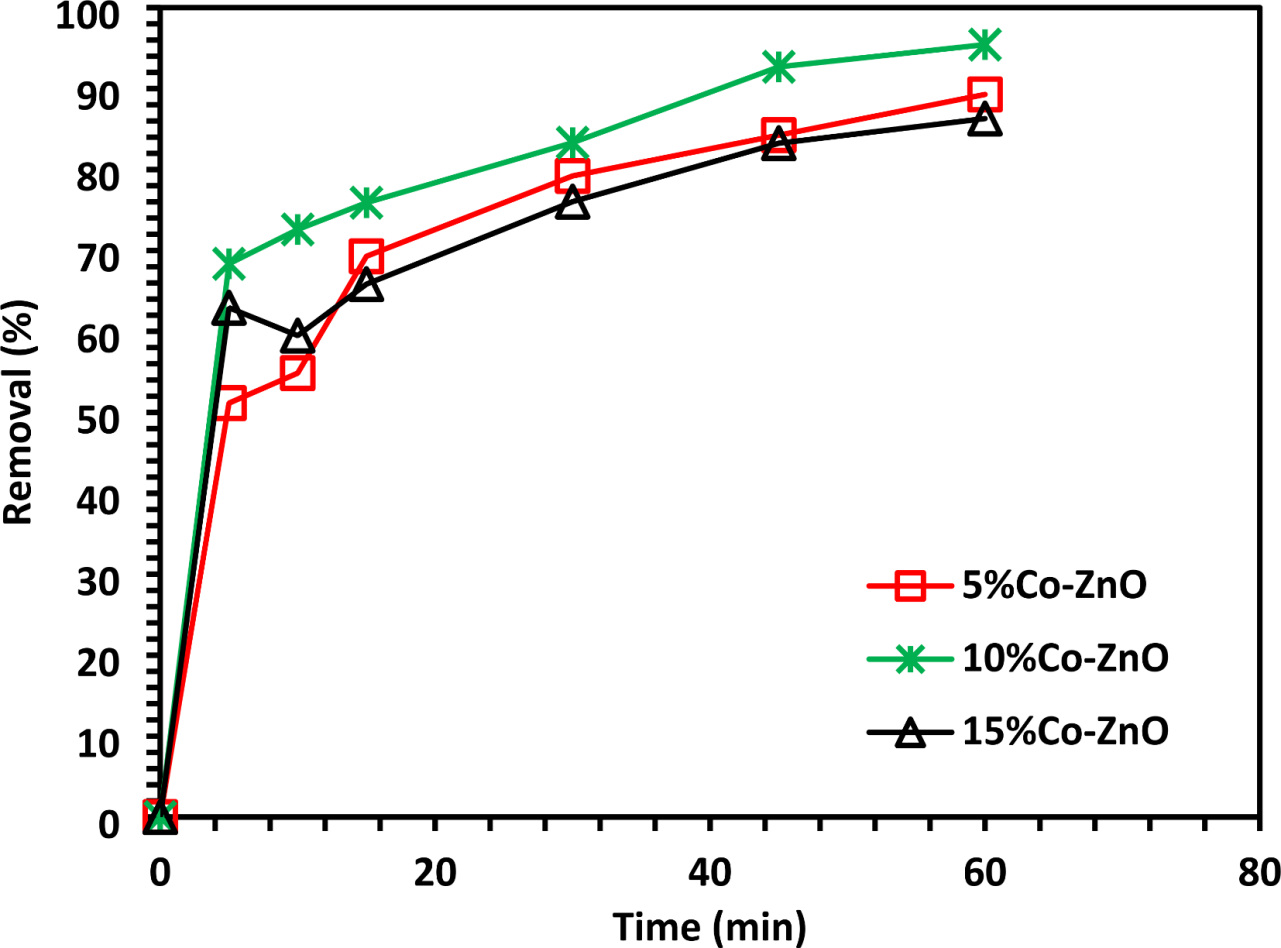
CIPF photocatalytic test of 5, 10, and 15% Co-ZnO NPs using CIPF 30 mg/L initial concentration and 20 mg/100 mL of catalyst doase.

The results indicate that after 60 min, 10% Co-ZnO NPs removed 95.4% of the CIB, which was the most outstanding performance (Fig. 10).

#### Point zero charge (pH_PZC_) of 10% Co-ZnO NPs

**Fig. 11:**
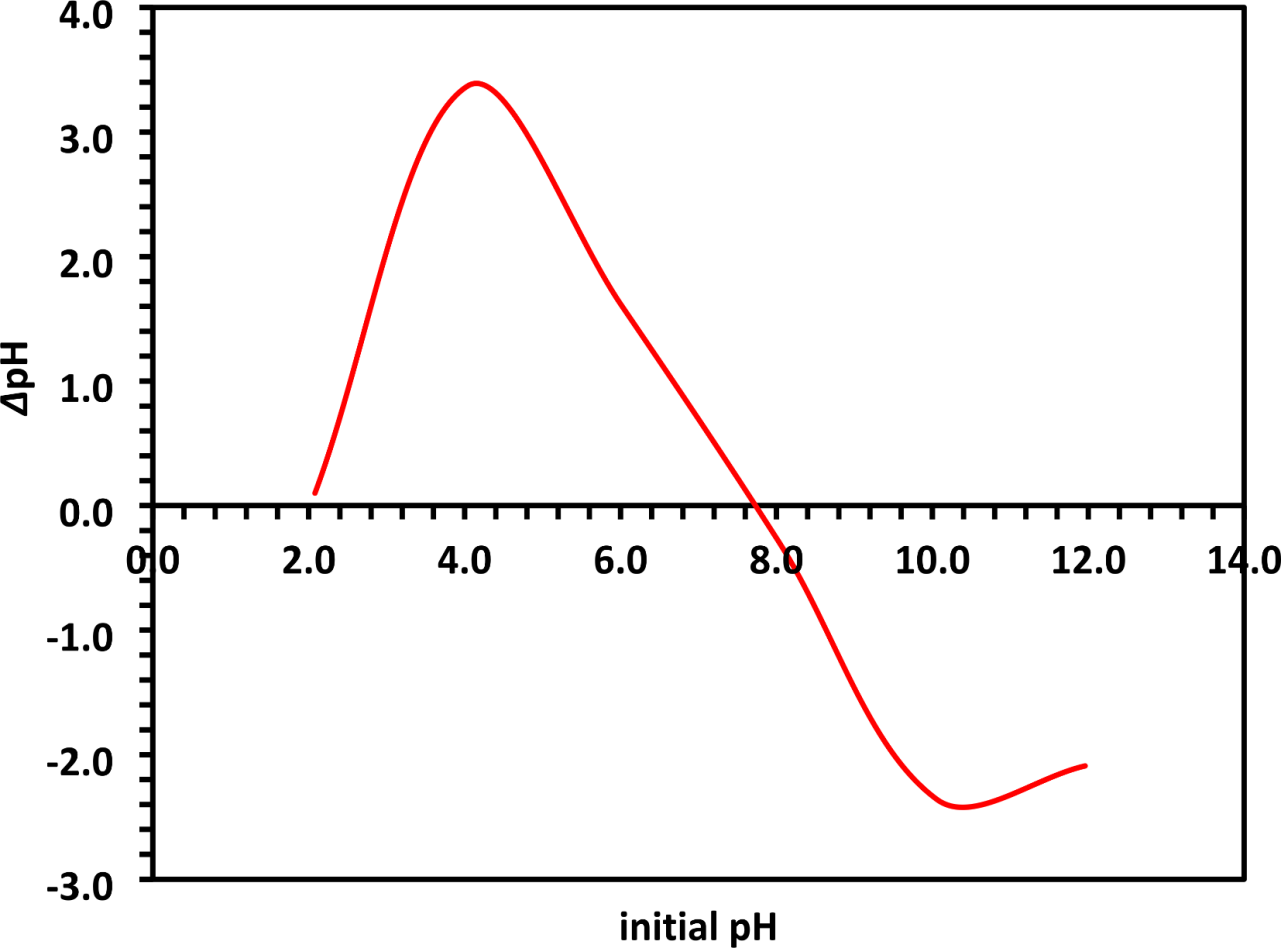
The pH_ZPC_ determination using 100 mg/100 mL of the 10% Co-ZnO NPs at initial pH values of 2, 4, 6, 8, 10, and 12.

The 10% Co-ZnO NPs pH_PZC_ was determined using an electrolyte solution of NaCl (0.1 N), from which the pH drift is measured. 0.1 N NaOH and HCl solutions were used to adjust the pH of the NaCl solution to 2, 4, 6, 8, 10, and 12. An equal volume of 0.10 g of 10% Co-ZnO NPs was mixed with 50 mL of the NaCl electrolyte solution. The solution control was placed on a mechanical shaker at 150 rpm for 24 h to reach pH equilibrium^35^. A histogram was drawn between the initial pH (2, 4, 6, 8, 10, and 12) and the final pH - the initial pH (*Δ*pH) after the interaction of the material with the electrolyte solution (Fig. 11). The point of intersection of the control curve and the curve with 10% Co-ZnO NPs gave the pH value pH_PZC_ of 10% Co-ZnO NPs^35^. Figure 11 shows that 10% Co-ZnO NPs have a pH of 7.8. This work demonstrates that the surface of Co-ZnO NPs has a negative charge above this pH due to the protonation of the functional groups, whereas the surface has a positive charge below this pH^35,37^. pH_PZC_ is an essential metric for determining the point on the surface of Co-ZnO NPs where the sites of positive charge and negative charge are equal. At that specific pH level, Co-ZnO NPs have a net zero charge on their surface, hence it may be considered that their surface charge is neutral. This results in a reduction of the isoelectric forces between ZnO NPs. It is possible to state that the surface charge is more favorable at pH values below pH_PZC_ and more damaging at pH levels above pH_PZC_^35,37^.

#### The pH impact on CIPF antibiotic photodegradation

**Fig. 12:**
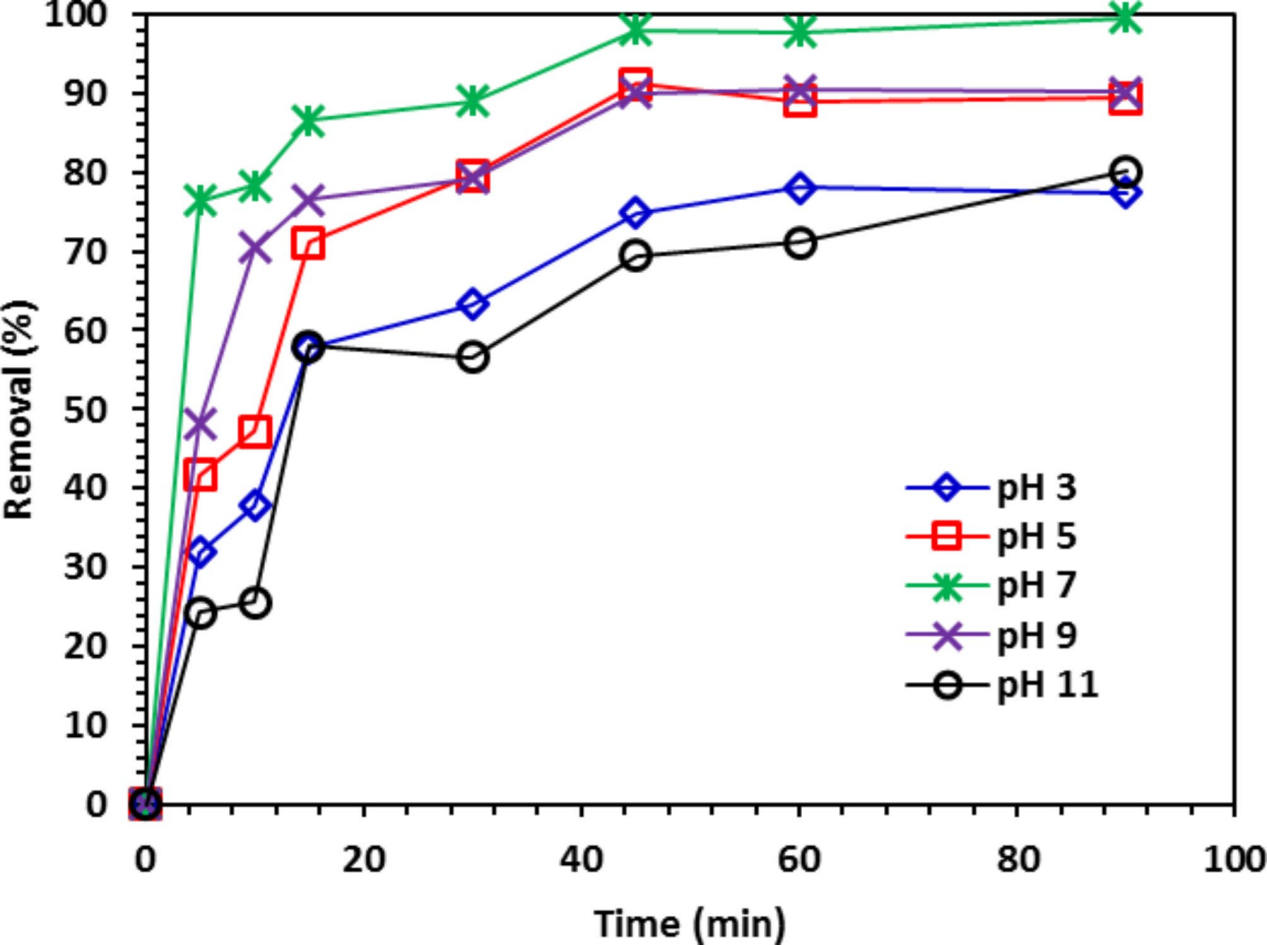
The impact of different pH values on the photo-degradation of CIPF (30 mg/L) in the existence of 10% Co-ZnO NPs (100 mg/100 mL at room temperature).

The adsorption effectiveness is significantly impacted by pH because a difference in pH induces changes in the ZnO NPs surface properties and CIPF ionization state^18^. The pH_ZPC_ for Co-ZnO NPs was discovered to be 7.8. So Co-ZnO NPs may have positive (+) or negative (−) charge depending on whether the solution’s pH is less than or greater than 7.8. As demonstrated in Fig. 12, the highest rate of CIP degradation on the Co-ZnO NPs happened at a pH of approximately 7. As previously reported, the dissociation constants for CIPF are pKa_1_ (for –COOH groups) = 6.1 and pKa_2_ (for –NH_2_ groups) = 8.7. At a pH under 6.1, the cationic state resulting from the amine group’s protonation dominates. The pH range (6.1–8.7) also contains the CIPF Zwitterion form. At pH 7, the maximum removal efficiency was 99%. At pH 7, the maximum removal efficiency was 99%. It is easier for electrostatic interaction to occur between positively charged sites of the Co-ZnO NPs catalytic surface and negatively charged atoms of the carboxyl groups in CIPF when the pH of the solution hits 7, where the Zwitterion form of CIPF is at its greatest concentration^14^.

#### The impact of 10% Co-ZnO NPs dosage on CIPF photodegradation

**Fig. 13:**
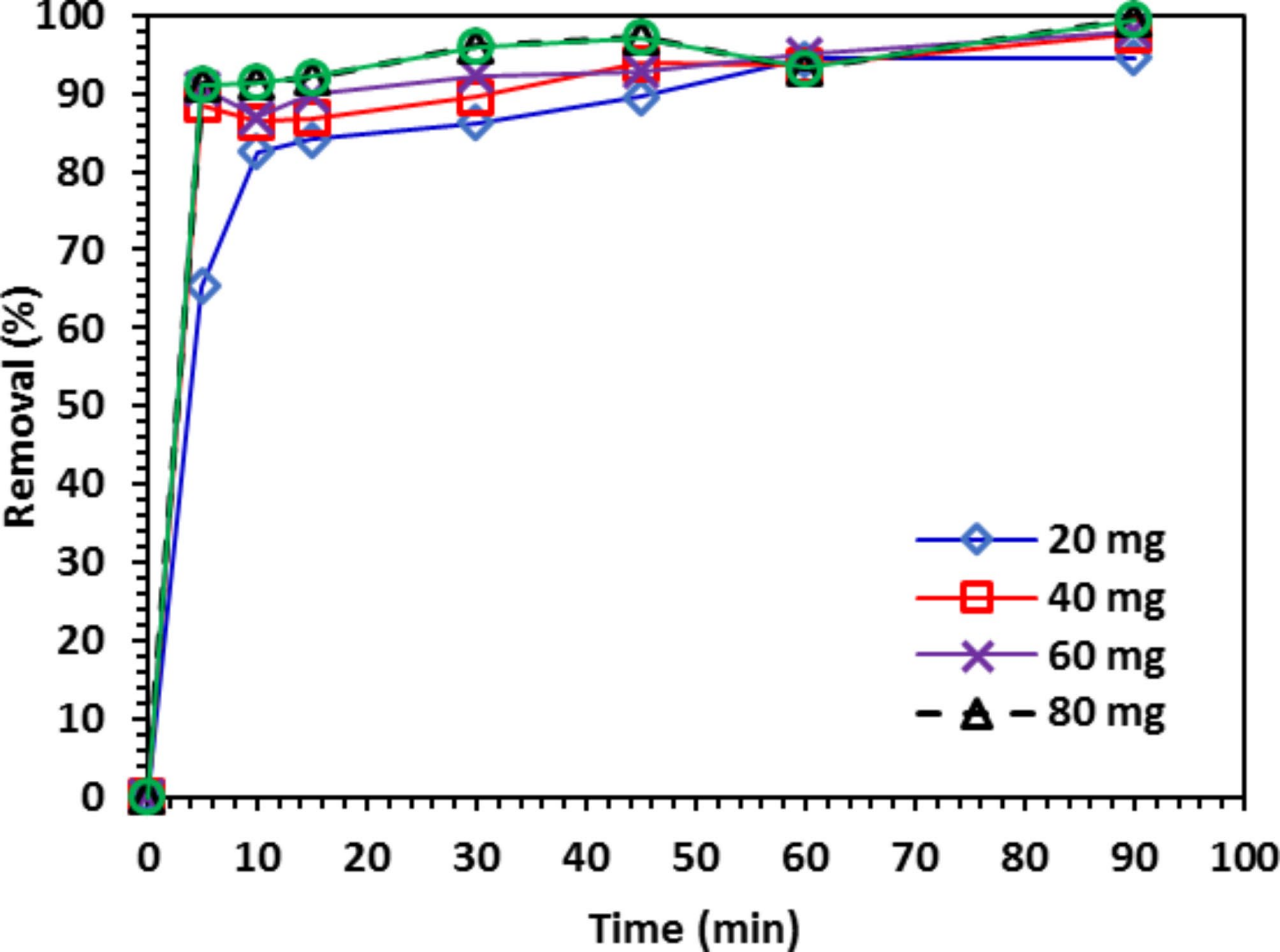
The impact of 10% Co-ZnO NPs (20–100 mg/100 mL) catalyst dosage on CIPF (30 mg/L) photo-degradation.

In the following experiment, the photocatalyst dosage was increased from 20 to 100 mg/100 mL and (30 mg/L CIPF initial concentration), while maintaining the solution pH = 7 at 25 °C and 200 rpm. A 10% Co-ZnO NPs catalyst dose ranged from 20 to 100 mg/100 mL, each given at a 20 mg interval. For various catalyst doses, such as 20, 40, 60, 80, and 100 mg/100 mL. The yield of the CIPF antibiotic was shown to be 94.5% and 99.8%, respectively, when the initial concentration of Co-ZnO NPs was 20 mg/100 mL and 100 mg/100 mL. Consequently, the initial concentration of Co-ZnO NPs directly impacts the removal efficiency. This may increase the number of active sites on the catalyst, subsequently increasing the formation of radical hydroxyls^38^. Increased nanoparticle concentration can be used to increase removal efficiency, as seen in Fig. 13. To boost the absorption and elimination of CIPF antibiotics, it is anticipated that as the catalyst’s concentration rises, more active sites will be accessible, and more free radicals will be generated. The catalysts stop the hydroxyl radical production and even prevent it from being used by upping the catalyst dosage. The increase in physical antibiotic absorption on the catalyst surface is caused by the catalyst acting as a sorbent and raising the catalyst dosage^48,49^.

#### The initial CIPF impact on its degradation process

**Fig. 14:**
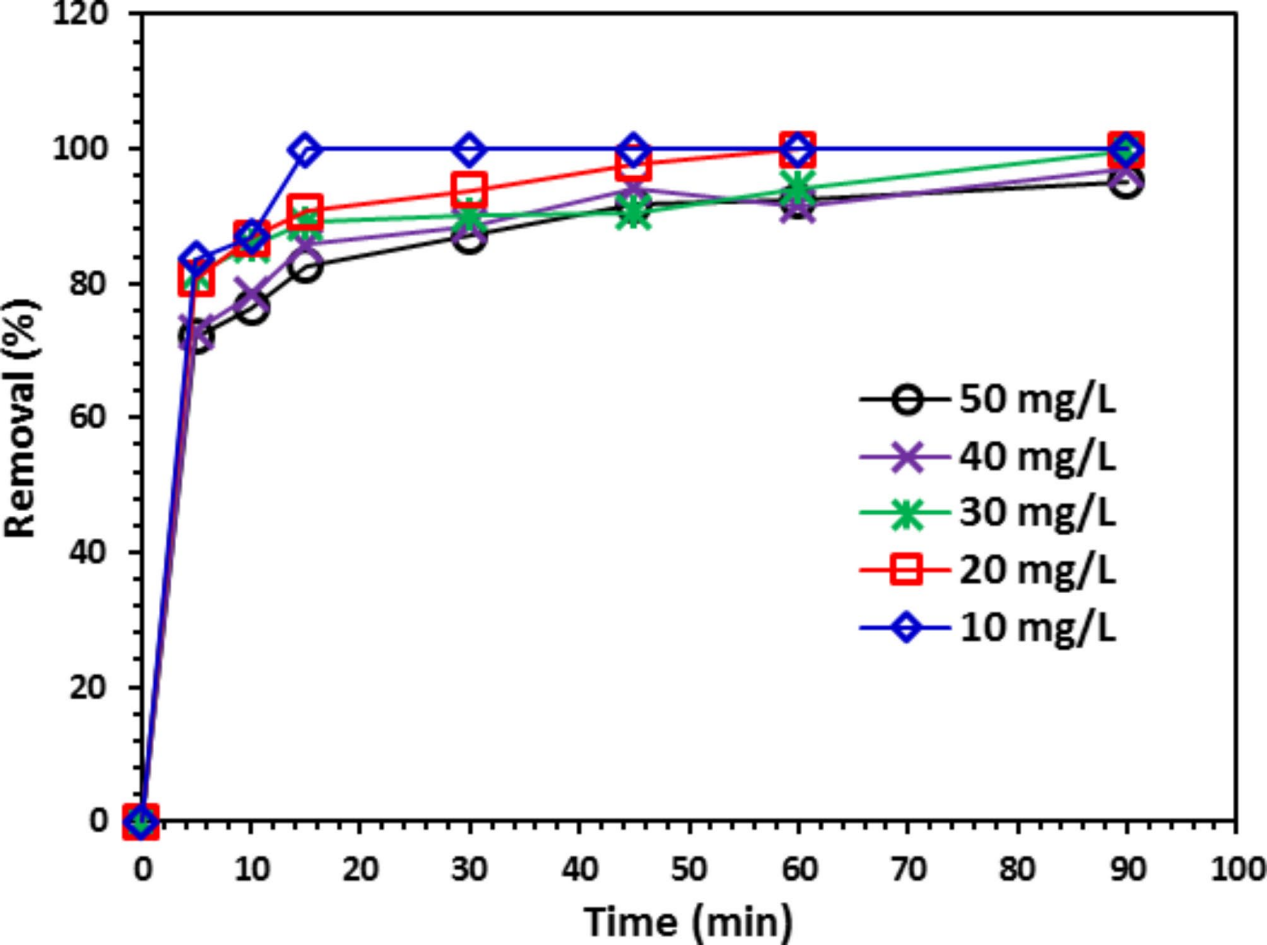
The CIPF antibiotic initial concentration impact on its degradation by 10% Co-ZnO NPs (1.0 g/L).

Figure 14 displays the findings of a study on the correlation between CIPF antibiotic initial concentration and time. The effectiveness of removal may be decreased if antibiotic concentrations are raised, it has been found. The most excellent antibiotic removal efficiency was attained at an initial dose of 10 mg/L; however, when the CIPF concentration increased to 50 mg/L, the removal efficiency declined. Because the number of active catalyst sites is fixed at constant ZnO concentrations, the removal efficiency tends to decrease with rising starting concentrations^45^. Raising the CIPF concentration causes the active sites to become saturated and the contaminating mole rate to increase, which lowers the efficacy of removal^49^.

#### The temperature effect on the degradation of CIPF

**Fig. 15:**
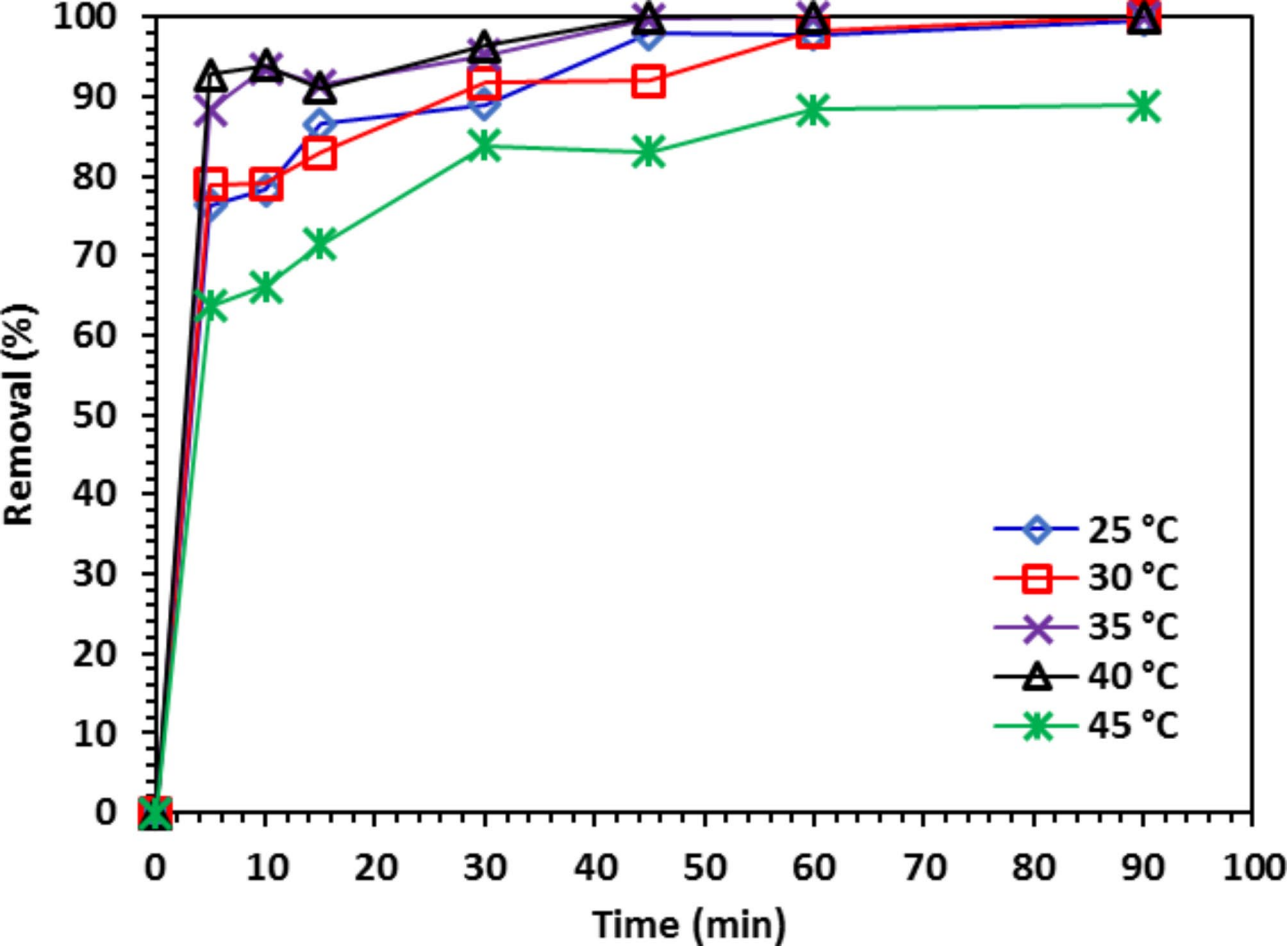
The 25, 30, 35, 40, and 45 °C temperature impact on the CIPF degradation (30 mg/L) using 10% Co-ZnO NPs (1.0 g/L).

At several temperatures (25, 30, 35, 40, and 45 °C), the photo-catalytic efficiency was examined to see how temperature impact on removal of CIPF from water using 10% Co-ZnO NPs. In photodegradation of CIPF increased from 25 to 40 °C, reaching its maximum removal at 40 °C (100%) in 45 min and then decreasing at 45 °C (Fig. 15). With increasing temperature, the increase in efficiency in the removal process is likely to follow Arrhenius’ law in which a temperature increase can increase particle mobility and the contact potential of catalyst particles with CIPF ions^50^. High temperatures impair the efficacy of the degradation process by causing the radicals to interact with one another rather than the CIPF molecule, which is why the minimal degradation efficiency was reached at these temperatures.

#### The effect of shaking speed on the degradation of CIPF

**Fig. 16:**
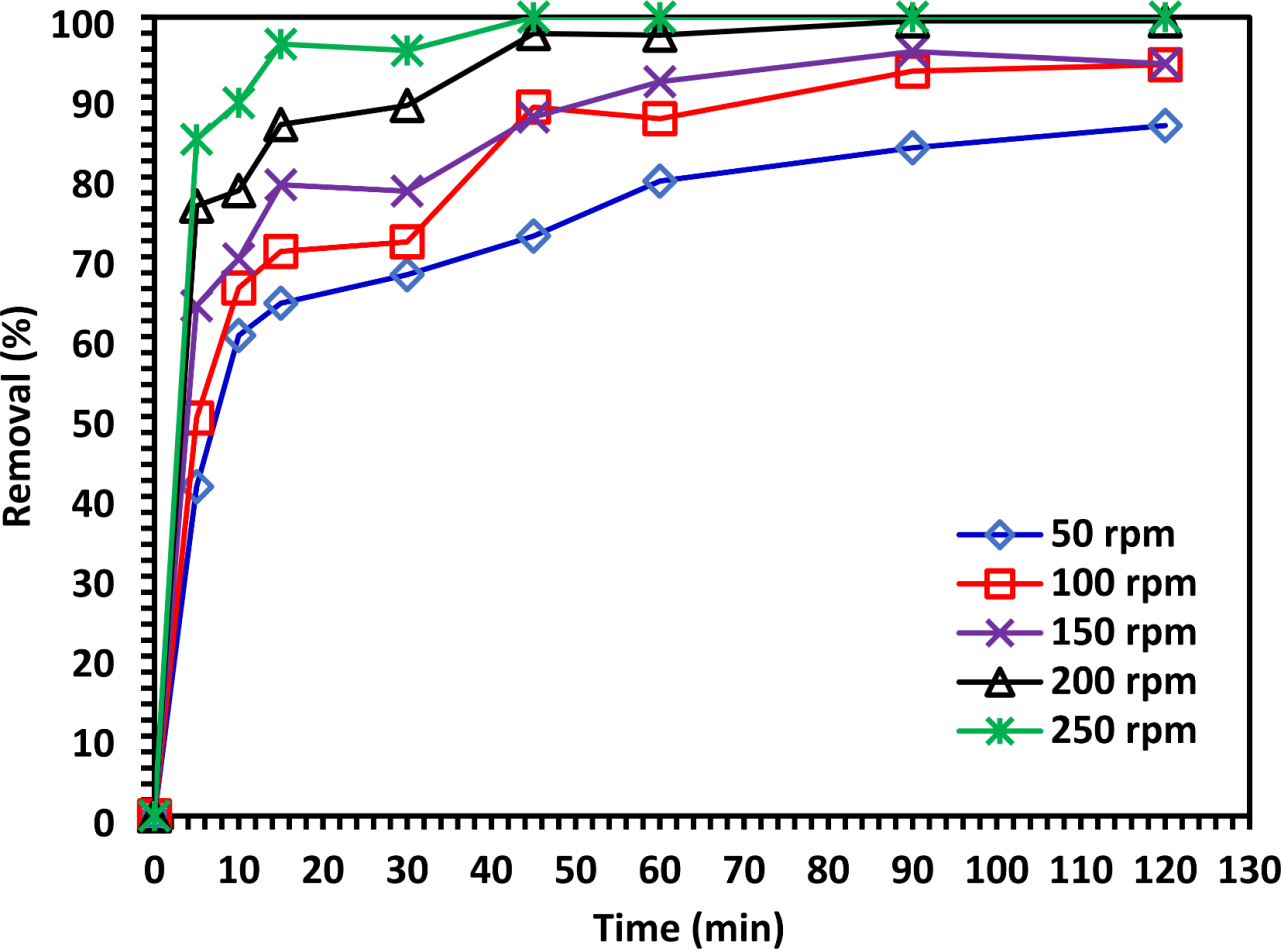
The shaking speed effect on the degradation of CIPF (30 mg/L) by 10% Co-ZnO NPs (1.0 g/L).

The removal effectiveness increased when the shaking speed rose from 50 to 250 rpm (Fig. 16). The boundary thickness around the adsorbent may have reduced as the shaking speed increased, which may have caused the degradation rate to increase. CIPF ion concentration would also rise close to the adsorbent surface^51^.

#### The scavengers’ effect on the CIPF removal efficiency

**Fig. 17:**
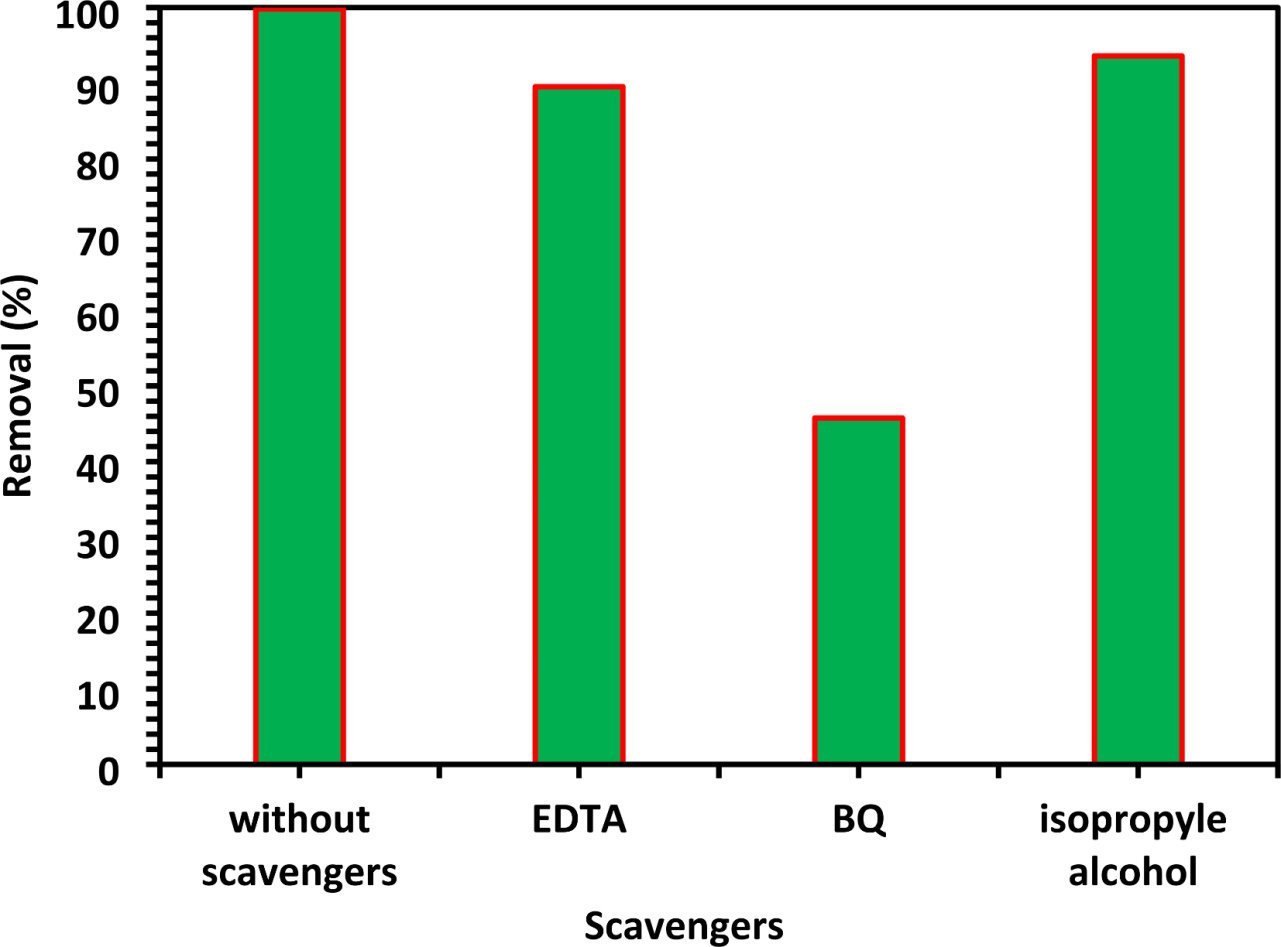
The effects of various scavengers on the CIPF photodegradation (30 mg/L) in the presence of a 10% Co-ZnO NPs catalyst (1.0 g/L) at pH = 7.

A typical active species that makes a considerable contribution to the photocatalytic degradation process is a hole, a hydroxyl radical, and a superoxide ion. To quench the hydroxyl radical (^**.**^OH), superoxide radical (O^2−^), and hole (h^+^), respectively, tests were conducted to capture active species by adding IPA (10 mM), BQ (1 mM), and Na-EDTA (10 mM)^11^. Without the addition of scavengers (Fig. 17), the 10% Co-ZnO NPs demonstrated that photocatalytic activity was achieved with 99.9% through 120 min of exposure to visible light. The photodegradation of CIPF is slightly affected by the Na-EDTA addition as a h^+^ scavenger, which dropped to 89.5% in the presence of 10% Co-ZnO NPs. The photodegradation of antibiotics did, however, significantly decline after the addition of IPA and BQ to 93.6 and 45.7%, respectively. As a result, the primary active species for CIPF degradation on Co-ZnO NPs under visible irradiation are OH and O_2_ radicals. Photocatalysis is expected to involve ^**.**^OH radicals containing a dual mechanism related to h^+^ and ^**.**^OH radicals^11,52^.

#### Kinetics of CIPF photocatalytic degradation

The photocatalytic degradation kinetic processes were described according to the first- and second-order kinetics. Equation (5) is used concerning the first-order, and Eq. (6) is used concerning the second-order kinetics (6)^10,50^.

5 $$\ln \left( {\frac{{C_{t} }}{{C_{0} }}} \right) = - k_{1} t$$

6 $$\frac{1}{{C_{t} }} = k_{2} t + \frac{1}{{C_{0} }}$$

**Fig. 18:**
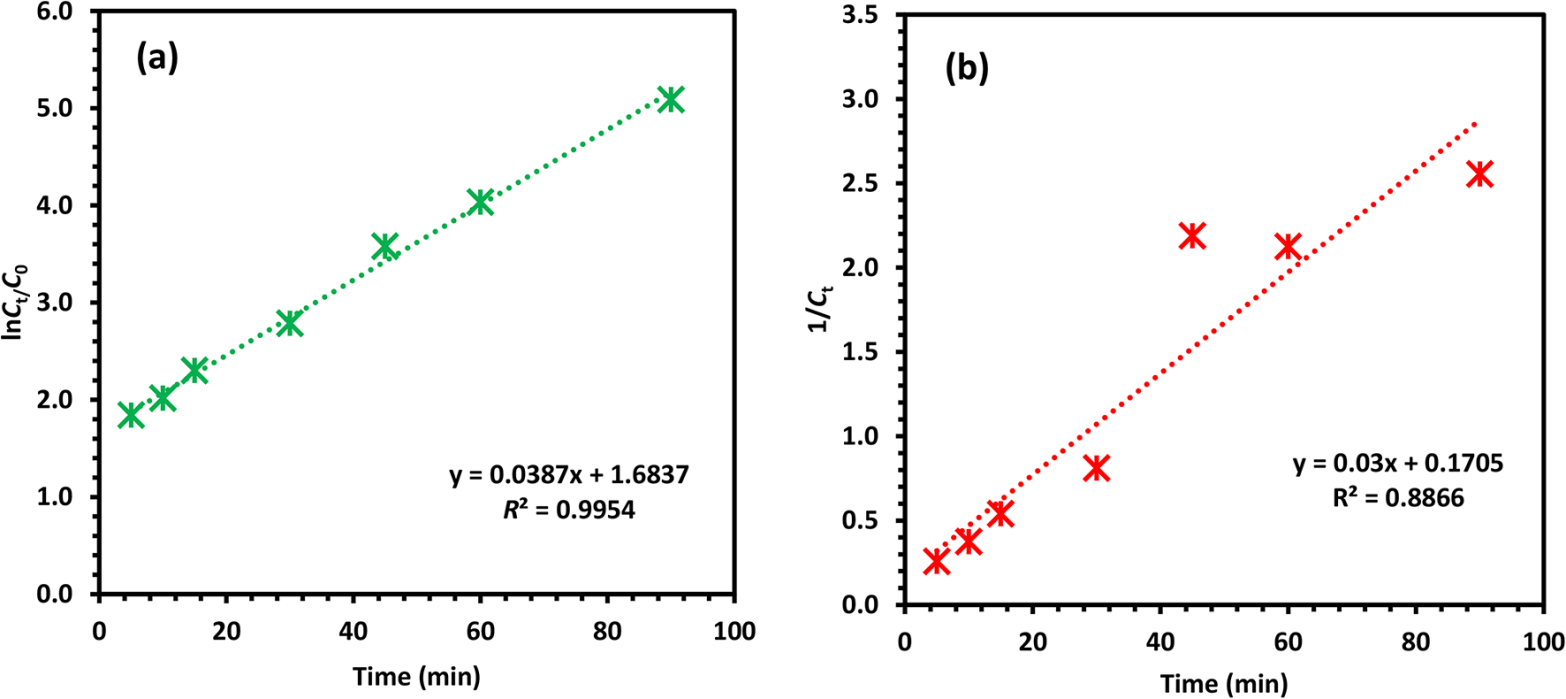
Kinetics of CIPF photo-catalytic degradation (**a**) first-order, (**b**) second-order kinetic models using 20 mg/L CIPF initial concentration at room temperature and from 0 to 90 min.

Based on a comparison of the regression coefficients (*R*^2^), the result is displayed in Fig. 18 as a CIPF photodegradation model employing the Co-ZnO NPs process. The reaction is recommended to follow first-order kinetics (*R*^2^ = 0.9954), while the second-order exhibited *R*^2^ = 0.8866. How rapidly photocatalytic reactions take place depends on the number of active sites on the catalyst surface and the quantity of contaminants. The reaction rates were found at various constant concentrations due to different catalytic breakdown processes or the conflict between intermediate products and reactive degradation^49^. First-order modelling is appropriate for displaying the response rate and CIPF elimination amount at any given time due to the linear relationship between CIPF concentration and exposure duration and the strong determination coefficient (*R*^2^)^52–56^.

#### Reusability of photocatalyst

**Fig. 19:**
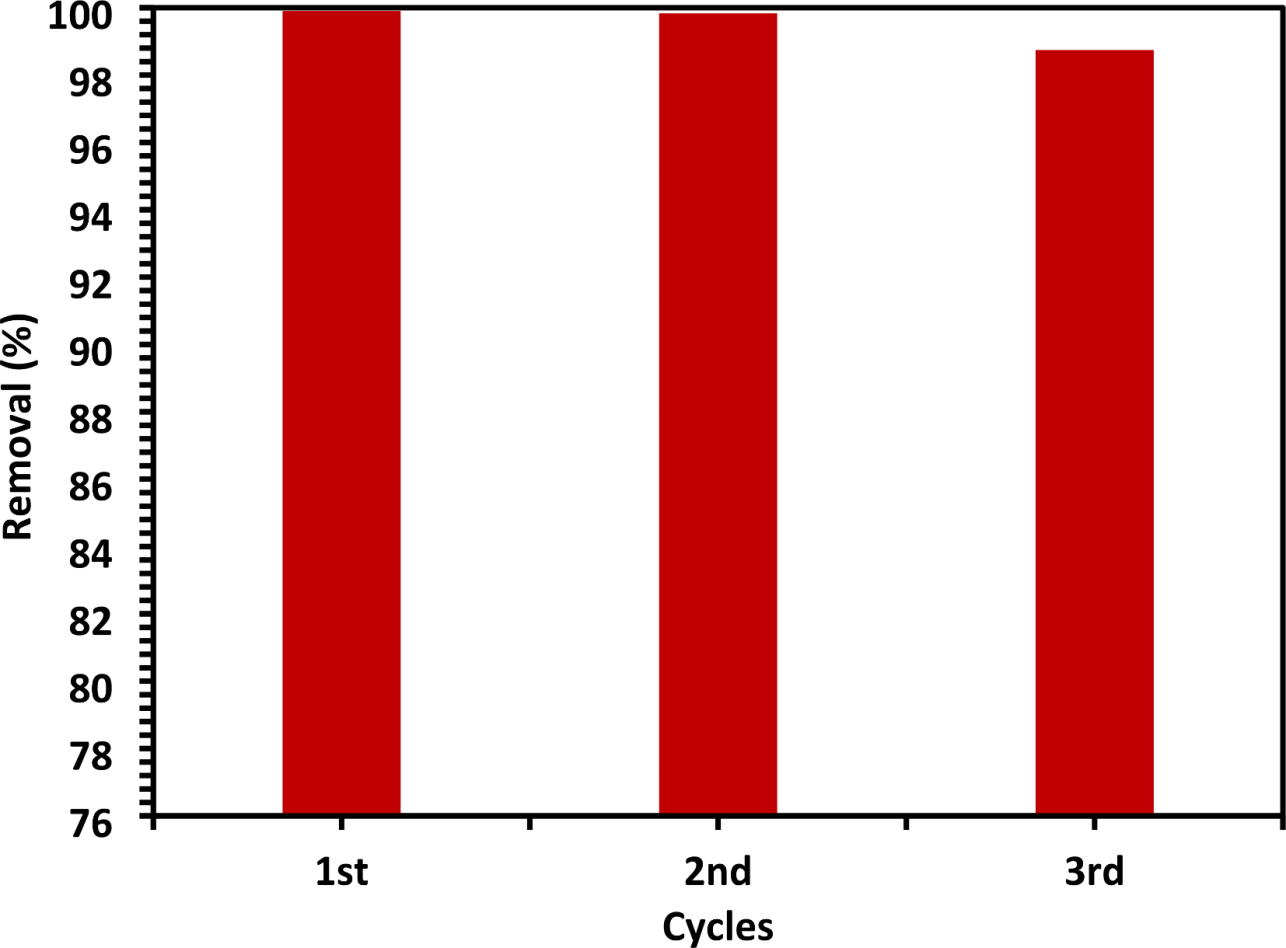
The CIPF (30 mg/L) photodegradation under visible light irradiation at room temperature may be recycled using 10% Co-ZnO NPs (100 mg/100 mL) in three successive cycles.

In optimum conditions for 10% Co-ZnO NPs, its reusability was investigated. The ability of 10% Co-ZnO NPs to degrade CIPF several times as a photocatalyst was examined. After the reaction, the Co-ZnO NPs were centrifuged for 25 min at 10,000 rpm, separated, and washed with EtOH and doubly distilled water three times before being preserved for the following reaction and then repeated this step twice^35^. CIPF removal efficiency occurred at concentrations of 30 mg/L (100 mL) with loadings of 100 mg of 10% Co-ZnO NPs, pH 7 at 25 °C, and 200 rpm. By using 10% Co-ZnO NPs more than times in the degradation of CIPF, tiny changes in removal efficiency decreased due to the decrease in the number of active sites for 10% Co-ZnO NPs. As presented in Fig. 19, for the first, second, and third cycles, the CIPF photocatalytic degradation using 10% Co-ZnO NPs as a catalyst reduced from 99.9 to 99.8 and 98.7%, respectively. Comparing the photocatalyst described in this work to previously published catalysts, the data in Table S4 indicates that the Co-ZnO 10% is efficient in photo-degrading of CIPF. The data in Table S4 shows that the photocatalyst reported in this study is more effective toward photodegradation of CIPF than already reported catalysts.

#### Proposed photodegradation mechanism

**Fig. 20:**
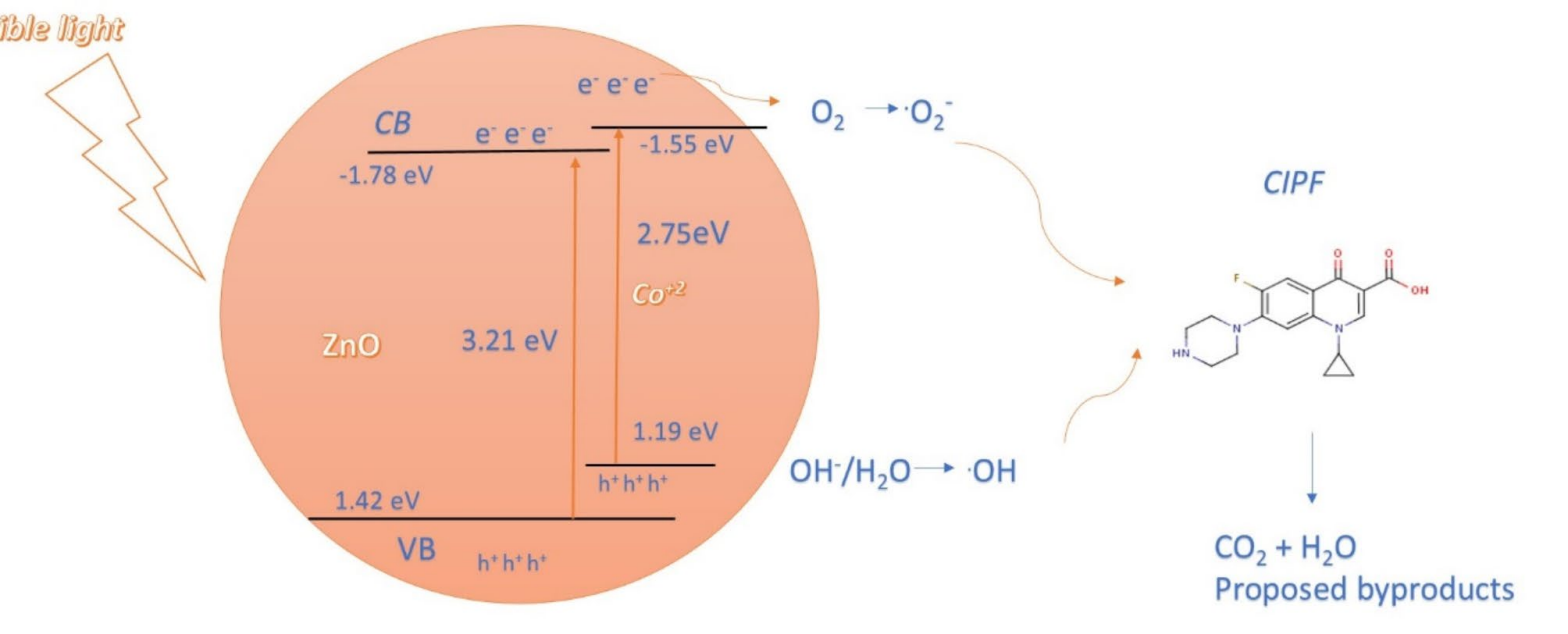
Photocatalytic activity mechanism of Co-ZnO NPs for the CIPF photodegradation at room temperature.

This is the suggested process for how pure ZnO and Co-ZnO NPs catalysts photocatalyze the breakdown of antibiotics CIPF. Electron-hole (e^−^/h^+^) pairs are created in ZnO as a result of the antibacterial photocatalyst being initially exposed to photons. In a redox process, the photogenerated e^-^/h^+^ couples interact with electrons in the ZnO NPs conduction band as they travel to the surface of the material. In another case, the antibiotic-adsorbed photocatalyst produces photogenerated e^−^/h^+^ pairs that move to the ZnO surface and go through a redox process, which interacts with the dissociation of water molecules to form ^**.**^OH radicals and electrons in the ZnO NPs conduction band. Co-ZnO NPs photocatalysts, which capture photo-excited electrons from the ZnO NPs conduction band, prevent electron-hole recombination. Co-ZnO NPs catalyst’s photocatalytic activity may be improved by increasing the lifetime of photo-generated charge carriers^13^. The decomposition of the CIPF molecules adsorbed on the catalyst is caused by a reaction between these two highly reactive radicals. The CIPF solution with catalysts present is also shown in Fig. 20 before and after being exposed to visible light. The succession of reactions that comprise the photocatalytic degradation process are as follows Eqs. (7–14) ^13,21^:

7 $${\text{ZnO}} + {\text{h}}\nu \to {\text{e}}^{-} ({\text{CB}}) + {\text{h}}^{ + } ({\text{VB}})$$

8 $${\text{e}}^{-} + {\text{O}}_{{2}} \to \bullet {\text{O}}_{{2}}^{-}$$

9 $${\text{Co}}^{{{2} + }} + {\text{e}}^{-} \left( {{\text{CB}}} \right) \to {\text{Co}}^{ + } \left( {{\text{electron}}\;{\text{trap}}} \right)$$

10 $${\text{Co}}^{ + } + {\text{O}}^{{2}} \to {\text{Co}}^{{{2} + }} + \bullet {\text{O}}_{{2}}^{-} \left( {{\text{electron}}\;{\text{release}}} \right)$$

11 $${\text{h}}^{ + } + {\text{OH}}^{-} \to \bullet {\text{OH}}$$

12 $${\text{h}}^{ + } + {\text{H}}_{{2}} {\text{O}} \to {\text{H}}^{ + } + \bullet {\text{OH}}$$

13 $$\bullet {\text{O}}_{{2}}^{-} + {\text{CIPF }} \to {\text{Degradation}}\;{\text{products}} + {\text{CO}}_{{2}} + {\text{H}}_{{2}} {\text{O}}$$

14 $$\bullet {\text{OH}} + {\text{CIPF}} \to {\text{Degradation}}\;{\text{products}} + {\text{CO}}_{{2}} + {\text{H}}_{{2}} {\text{O}}$$

#### CCD optimization results

Table 4 provides the experimentally determined results and model-predicted values. This demonstrates how the different amounts affect the degradation of antibiotics. According to the model analysis, Eq. (15) was utilized to build a quadratic response surface to explain the relationship between the independent variables and experimental results:

15 $$\begin{aligned} {\text{CIPF}}\;{\text{degradation}}\left( \% \right) & = {94}.0{1} + 0.{\text{6576A}} + {5}.{5}0{\text{B}} + 0.0{\text{279C}}{-}0.{4}0{\text{29D}}{-}0.{\text{2591AB}} \\ & \quad + 0.0{\text{512AC }} + \, 0.{\text{5943AD }} + { 1}.{\text{16BC}}{-}{1}.{\text{62BD }} + \, 0.{\text{7339CD}} \\ & \quad {-}0.{\text{1535A}}^{{2}} {-}{2}.{\text{96B}}^{{2}} {-}{1}.{\text{42C}}^{{2}} {-}{6}.{\text{49D}}^{{2}} \\ \end{aligned}$$

**Table 4:** The central composite design table for CIPF degradation includes intended values, experimental and anticipated outcomes.

Run	A 10% Co-ZnO NPs dosage (mg)	B CIPF dosage (mg/L)	C Shaking speed (rpm)	D pH	Removal actual value	Removal predicted Value
1	60	30	250	7	92.486	88.40
2	100	30	150	7	94.174	94.71
3	40	40	200	9	89.250	87.34
4	60	30	150	7	94.009	94.01
5	80	20	200	5	64.846	74.77
6	80	40	100	5	86.271	89.82
7	80	20	200	9	87.190	79.87
8	80	20	100	5	79.990	78.40
9	60	30	150	11	51.194	67.24
10	60	30	150	7	94.009	94.01
11	60	30	150	7	94.009	94.01
12	80	40	200	9	91.063	89.43
13	40	20	200	5	73.486	74.02
14	40	40	100	5	86.495	90.31
15	40	40	100	9	91.087	83.60
16	60	30	150	7	94.009	94.01
17	40	40	200	5	85.079	91.12
18	60	50	150	7	94.161	93.17
19	60	30	150	7	94.009	94.01
20	20	30	150	7	91.559	92.08
21	60	30	50	7	83.151	88.29
22	80	20	100	9	84.161	80.56
23	60	30	150	7	94.009	94.01
24	60	10	150	7	69.116	71.16
25	40	20	100	9	83.714	77.64
26	40	20	100	5	73.784	77.86
27	80	40	100	9	89.523	85.49
28	80	40	200	5	88.257	90.83
29	60	30	150	3	83.846	68.85
30	40	20	200	9	77.855	76.75

The equation provided in terms of coded factors (CF) can be used to anticipate the reaction for the particular concentrations of each element. By default, + 1 represents the high values of the components and −1 represents the low levels. Utilize the coded equation to compare the factor coefficients and ascertain the respective weights of the components.

Tables S5–S6 display the results, indicating that the model is significant, as indicated by the Model *F*-value of 2.52. There is only a 4.30% chance that an *F*-value of this size will be caused by noise^57,58^. P-value is a helpful tool for identifying the pattern of variable interactions^38,59^. The high *P*-value for the model (0.043) and the negligible *P*-value for lack of fit show that the quadratic model was appropriate for this investigation. The computed *R*^2^ was 0.7019, indicating that the model can account for 70.19% of the variance in the data. The expected *R*^2^ was -0.7171 while the adjusted *R*^2^ was 0.4237. The overall average may be a stronger predictor of response than the present model, according to a negative projected *R*^2^ for the model. Given that Adeq Precision assesses the signal-to-noise ratio, a higher-order model may occasionally provide superior predictions. The ideal ratio is greater than 4. Your ratio of 5.01 indicates a sufficiently strong signal. The result of PRESS (predicted residual error sum of square = 5198.50), which indicates that the model can match the data, suggests that the model may be utilized to explore the design space^38,59^. The link between antibiotic degradation and individual factors may be expressed using the second-order polynomial equation, as Table S6 shows how each parameter influences the degradation.

The residuals are shown to be very near to the diagonal line by the normal probability plot (Fig. S4a). This demonstrates that the model could adequately describe the link between the tested variables and the findings because the plot reflects the independence of the residuals^57,60^. Good consistency between these two parameters is shown by the graphical display of the anticipated data vs. the experimental data (Fig. S4b). The residual values are distributed randomly between −4 and + 4 on the graphs of residual vs. anticipated values (Fig. S4c). Additionally, the Box-Cox plot confirms the model’s strong predictability, and the model response does not require alteration (Fig. S5).

The perturbation plot, which is designed to look into the simultaneous effects of four factors on antibiotic elimination, is shown in Figure S6. This figure examines each component’s impact at a specific point in the design space^38,60^. The antibiotic concentration (B), shaking rate (C), pH level (D), and catalyst dosage (A) were the parameters controlling the breakdown of antibiotics. The antibiotic concentration (B) curve suggests this component is more important than the others.

**Fig. 21:**
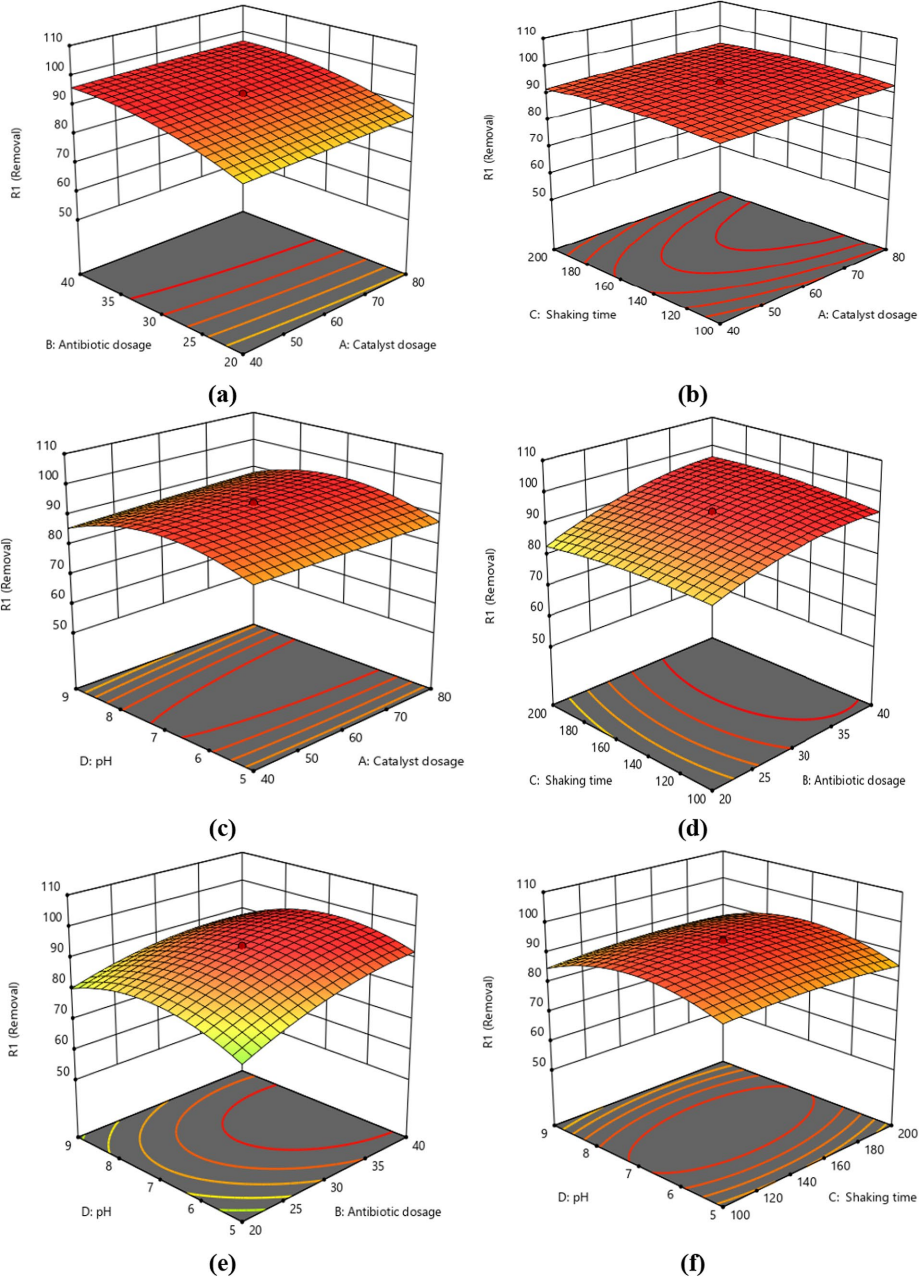
The following are three-dimensional response surface plots: (**a**) dosage of antibiotics and catalyst; (**b**) dosage of shaking speed (rpm) and catalyst (mg); (**c**) pH value and catalyst dosage (mg); (**d**) dosage of CIPF (mg/L) and shaking speed (rpm); (**e**) pH value and CIPF dosage (mg/L); and (**f**) pH value and shaking speed (rpm).

The ideal condition is better understood by looking at the 3D surface plots in Fig. 21, which show various forms of interactions between the independent variables. By fixing two of the four parameters, the second-order polynomial equation shown in this figure can be seen^60–67^. In contrast to the catalyst dose, which dropped from 0.2 to 0.1 g/L, the antibiotic concentration changed from 10 to 50 mg/L, as can be shown. 150 rpm was used for shaking, and 7 was the pH level.

The regression equation visually represented by the 2D contour plots and 3D surface response plots was used in the current investigation to estimate the optimal amounts of various elements. Usually, these plots are employed to comprehensively comprehend the interplay of the many aspects of the response. The 3D surface response, the 2D contour plots, and the outcomes of the interactions between the four components are displayed in Figs. 21 and S7. Figure 21a illustrates how raising CIPF concentration and photocatalyst dose improves CIPF’s photodegradation effectiveness. Figure 21b illustrates how the dosage of the photocatalyst and the rate of shaking interact to affect the photodegradation effectiveness of CIPF, Where the photodegradation rate increased with increasing shaking speed and photocatalyst dosage amount. According to Fig. 21c, the photodegradation rate rose as the photocatalyst dose amount and pH changed from 5 to 7 and then decreased from 7 to 9, respectively. The photodegradation effectiveness of CIPF is shown in a 3D plot in Fig. 21d as a function of CIPF concentration and shaking rate, with the photocatalyst dose and pH remaining constant at 0.06 g/L and 7, respectively. According to the findings, CIPF degrades less quickly when its concentration is high. Figure 21e explains the interaction impact of antibiotic dosage and pH on the degradation of CIPF while maintaining photocatalyst concentration and shaking rate at their optimum values of 60 mg and 150, respectively. The results indicated that increasing the CIPF concentration and increasing the pH up to 7 leads to increased degradation of CIPF, which decreases to higher than 7. In Fig. 21f, the relations between pH and shaking speed on the degradation efficiency of CIPF shows that photodegradation increased with increasing shaking speed and pH from 5 to 7 and then down from 7 to 9.

**Fig. 22:**
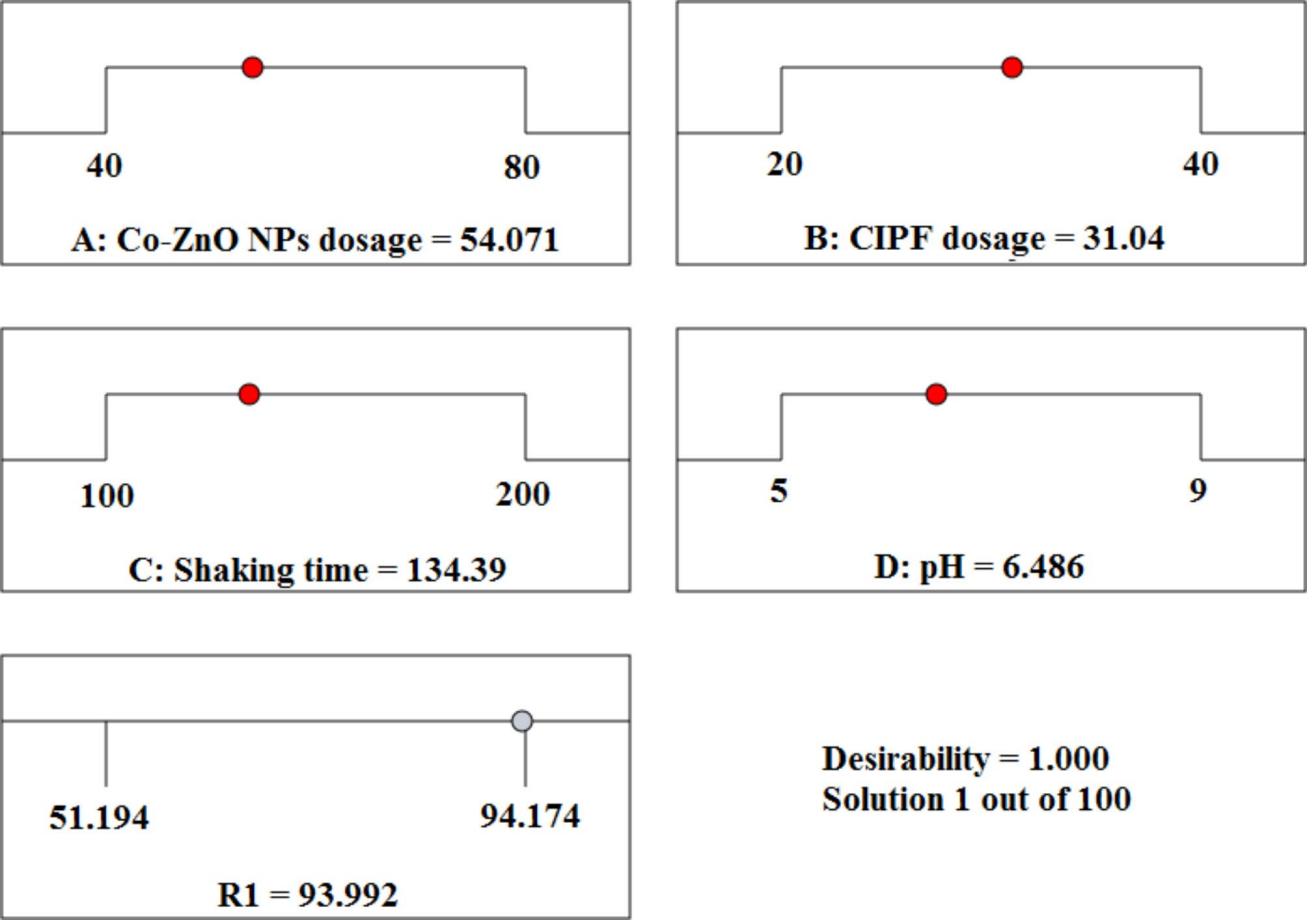
Optimized settings for a CCD-based final answer of solution 1 out of 100 with desirability equal to 1.

An optimization test was run to determine the best operational setting for maximizing efficiency. As a result, the proposed model with the parameters shaking speed of 134.32, catalyst dose = 54.071 mg, pH 6.487, and antibiotic concentration of 31.04 mg/L obtained the most significant degradation of CIPF (93.992%). A description of the findings is shown in Fig. 22. The experimental CIPF degradation was 94.17%, confirming the model’s accuracy in forecasting process efficiency using operational variables synthesized at different scales.

#### ANN modelling

**Fig. 23:**
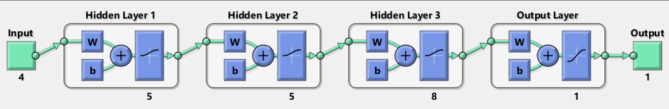
ANN architecture for the removal of antibiotic ciprofloxacin.

**Fig. 24:**
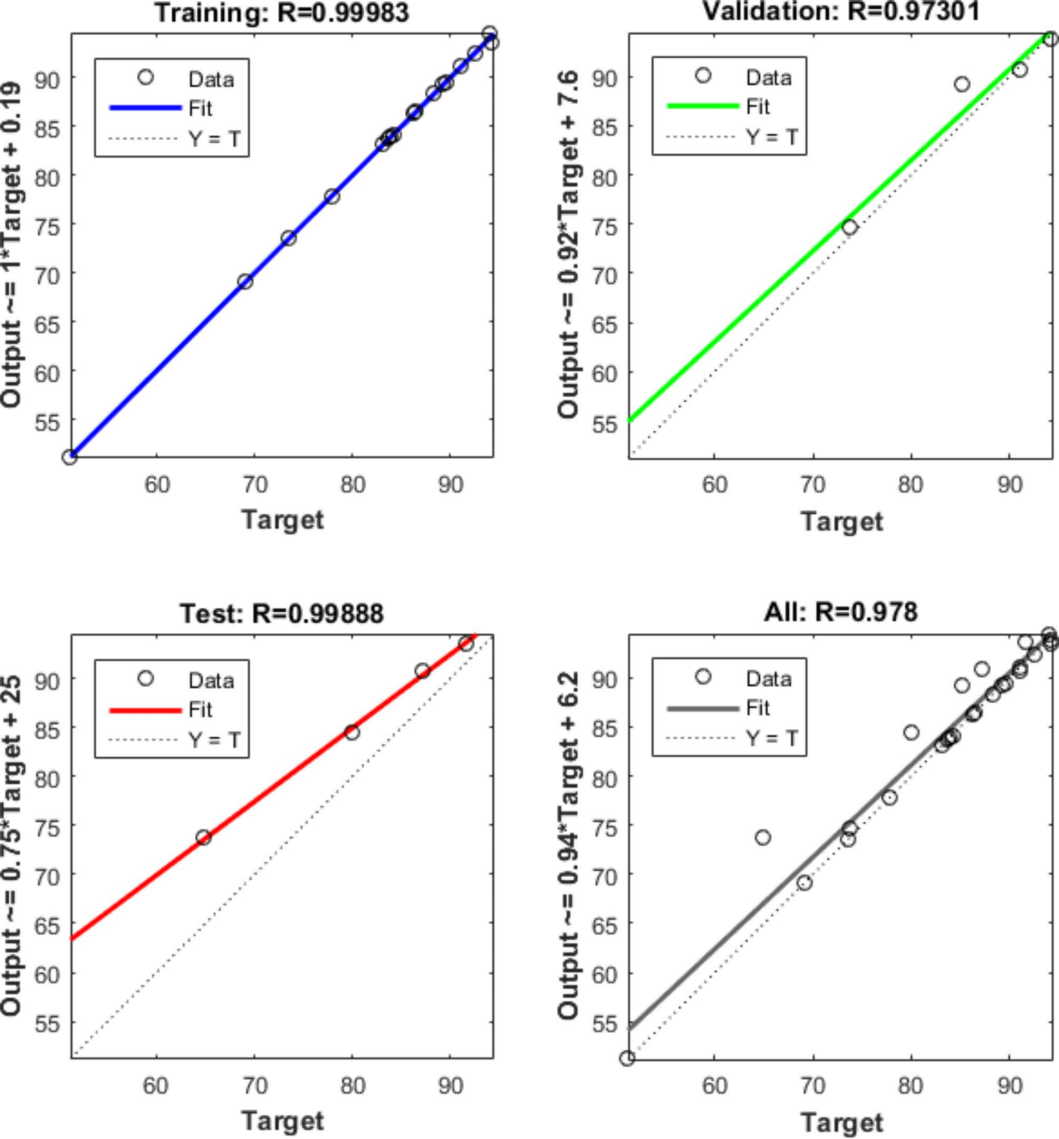
Training, validation, testing, and overall datasets for the LM algorithm.

**Fig. 25:**
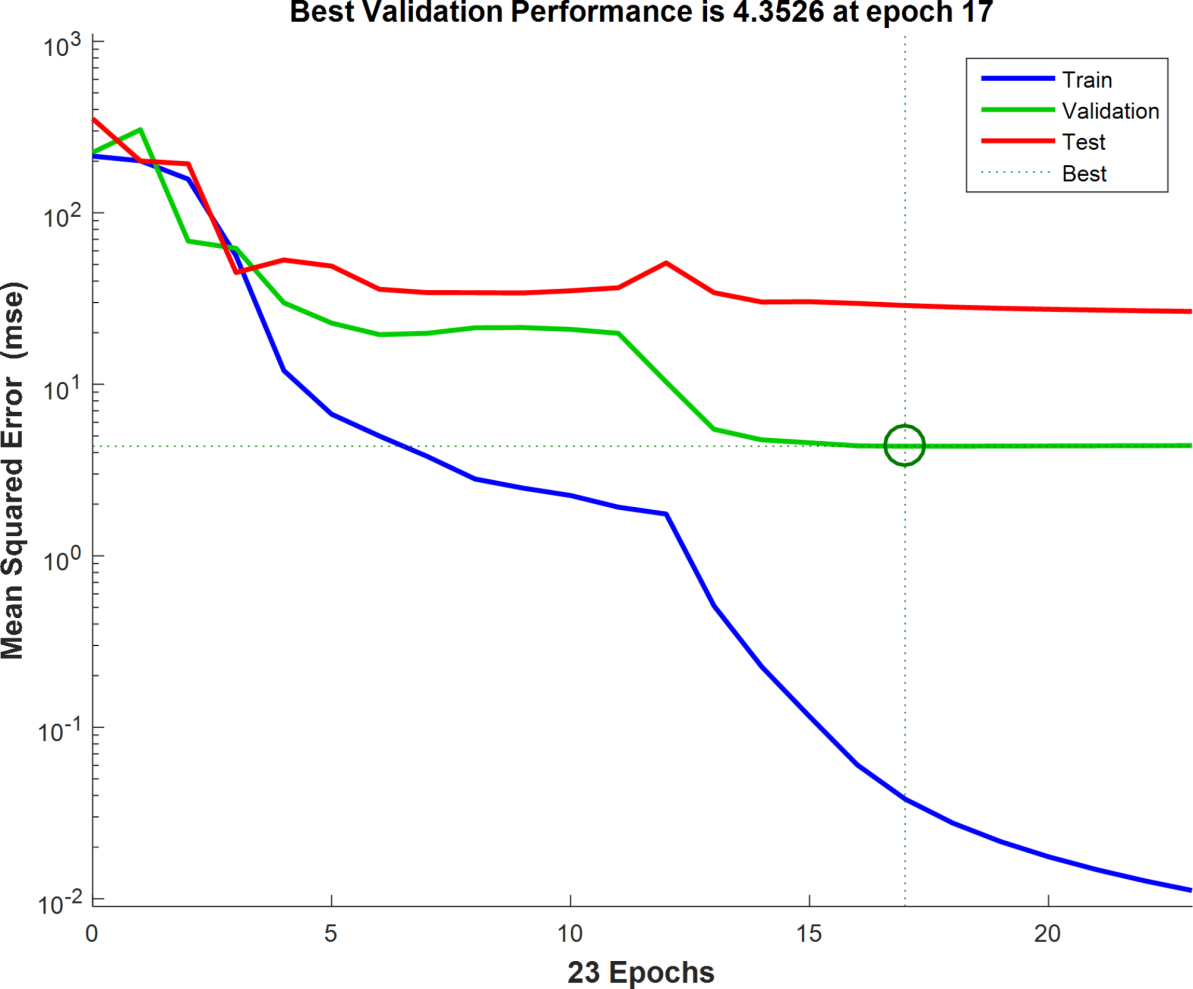
LM algorithm performance.

The classification of the sample data in this study is 70% training, 15% validation, and 15% testing. The ANN performance, ANN performance gradient, validation checks number, and training epoch number were the most critical factors for ANN training. The best fit ANN model properties are the highest *R*^2^ and the least MSE. The best ANN training procedure was the backpropagation algorithm (trainlm). The best-fit network for the photodegradation removal of antibiotic ciprofloxacin using fabricated cobalt-doped zinc oxide nanoparticles as a catalyst was found as 4-5-5-8-1 (4- ILs, 5- HL 1, 5-HL 2, 8-HL 3, and 1 OL) as shown in Fig. 23. The regression plots are shown in Fig. 24. The *R*^2^ for training, validation, testing, and overall were 0.9998, 0.9730, 0.9989, and 0.978, respectively. The MSE value was 0.112. The low MSE value and high *R*^2^ in this study proved that the performance of the ANN model was perfect although outliers. This study contained 4 input variables (the Catalyst dosage of the fabricated cobalt-doped zinc oxide nanoparticles (mg), shaking speed (rpm), pH, and antibiotic dosage of ciprofloxacin (mg)) and 1 output variable (photodegradation removal of antibiotic ciprofloxacin). The best NN activation functions were Log-Sigmoid (log-sig) and Tan-Sigmoid (Tan-sig) for the 1^st^ and 2^nd^ hidden layers. Purelin is the activation function for the 3^rd^ hidden layer and output layer. Figure 25 shows the MSE error vs the epoch number for the optimized ANN model. The best NN training process stopped after 17 epochs. This result meant that the ANN training model was flawlessly performed at 17 for modelling the adsorption process^68^.

## Energy consumption and economic analysis

The study’s findings provide valuable insights into the energy consumption and economic feasibility of using 10% Co-ZnO nanoparticles (NPs) as photocatalysts for ciprofloxacin (CIPF) degradation. Using visible LED light, the process achieved up to 94% degradation under optimal conditions (54.071 mg of NPs, 31.04 mg/L initial CIPF concentration, pH 6.48, and 134.39 rpm) within 90 min. This setup ensures energy-efficient operation since visible LED light consumes less energy than other light sources like UV lamps, making the process more sustainable and cost-effective. From an economic perspective, the precise optimization of reaction parameters minimizes the amount of catalyst and energy required, reducing operational costs. A predictive ANN model with a high correlation coefficient (R^2^ = 0.978) further enhances efficiency by allowing accurate simulation and planning, avoiding unnecessary trials and material wastage. The scalable and energy-efficient nature of the process, coupled with the high degradation rates, suggests its potential for cost-effective implementation in wastewater treatment systems. However, a detailed cost-benefit analysis, including the production cost of Co-ZnO NPs and long-term operational expenses, would be essential for assessing the entire economic viability of this technology.

## Conclusions

This study focuses on producing and testing ZnO and Co-ZnO nanoparticles (NPs) with 5%, 10%, and 15% cobalt doping as photocatalysts for breaking down the antibiotic ciprofloxacin (CIPF). X-ray diffraction (XRD) analysis revealed that the NPs had a hexagonal wurtzite structure and an average size between 38.47 and 48.06 nm. Among them, 10% Co-ZnO NPs showed the best performance, degrading 99% of CIPF in 90 min under visible light. The degradation process followed first-order kinetics. An optimization CCD model was applied to test the photocatalytic activity of 10% Co-ZnO NPs. Under ideal conditions (a dose of 54.071 mg of NPs, initial CIPF concentration of 31.04 mg/L, pH 6.48, and 134.39 rpm), the removal rate reached 94% after 90 min using visible LED light. The experimental results were also analyzed using artificial neural networks (ANN). A network with three hidden layers (5, 5, and 8 neurons in each layer) provided the most accurate predictions, achieving a correlation coefficient (R^2^) of 0.978. This indicates the model can effectively predict photocatalytic performance.

## Electronic supplementary material

Below is the link to the electronic supplementary material.

Supplementary Material 1

## Data Availability

The datasets used in this investigation are accessible for review upon request from the paper’s corresponding author.
