# Supplementary material for: Kinetics, central composite design and artificial neural network modelling of ciprofloxacin antibiotic photodegradation using fabricated cobalt-doped zinc oxide nanoparticles
Source: Sci Rep. 2025 Jan 10;15:1610. doi: 10.1038/s41598-024-84568-w (PMC11724027; doi:10.1038/s41598-024-84568-w)
Supplement: Supplementary file 1 — Supplementary Material 1 [file 41598_2024_84568_MOESM1_ESM.docx]

**Supplementary materials**

**Kinetics, Central composite design and Artificial Neural Network modelling of ciprofloxacin antibiotic photodegradation using fabricated cobalt-doped zinc oxide nanoparticles**

Asmaa I. Meky^1^, Mohamed A. Hassaan^2^, Mohamed A. El-Nemr^3^, Howida A. Fetouh^1^, Amel M. Ismail^1^, Ahmed El Nemr^2^*

^1^Department of Chemistry, Faculty of Science, Alexandria University, Alexandria, Egypt

^2^Environment Division, National Institute of Oceanography and Fisheries (NIOF), Kayet Bey, Elanfoushy, Alexandria, Egypt

^3^Department of Chemical Engineering, Faculty of Engineering, Minia University, Minia 61519, Egypt.

Email: [asmaameky16@gmail.com](mailto:asmaameky16@gmail.com) (A. Meky), [mhss95@mail.com](mailto:mhss95@mail.com) (M.A. Hassaan), [mohamedelnemr1992@yahoo.com](mailto:mohamedelnemr1992@yahoo.com) (M.A. El-Nemr), [amelmostafa@yahoo.com](mailto:amelmostafa@yahoo.com) (A.M. Ismail), [howida_fetouh@alexu.edu.eg](mailto:howida_fetouh@alexu.edu.eg) (H.A. Fetouh).

*Corresponding author: [ahmedmoustafaelnemr@yahoo.com](mailto:ahmedmoustafaelnemr@yahoo.com); [ahmed.m.elnemr@gmail.com](mailto:ahmed.m.elnemr@gmail.com)

**Table S1**. Analysis of the surface area of ZnO NPs and Co doped ZnO NPs samples.

|  | | ZnO | 5% Co-ZnO | 10% Co-ZnO | 15% Co-ZnO |
| --- | --- | --- | --- | --- | --- |
| BET | *a*_s, BET_ (m^2^∕g) | 7.325 | 37.876E | 32.033 | 32.855 |
|  | *V*_m_ (cm^3^ STP)/g) | 1.683 | 8.7022 | 7.3597 | 7.5485 |
|  | Mean pore diameter *P*_m_ (nm) | 11.05 | 16.280 | 13.817 | 18.381 |
|  | Volume of total pore *V*_T_ (cm^3^/g) | 0.02023 | 0.1542 | 0.1106 | 0.1510 |
| BJH | *V*_p_ (cm^3^/g) | 0.02048 | 0.1542 | 0.1112 | 0.1517 |
|  | *a*_p_ (m^2^/g) | 7.9095 | 39.583 | 34.278 | 35.393 |

| 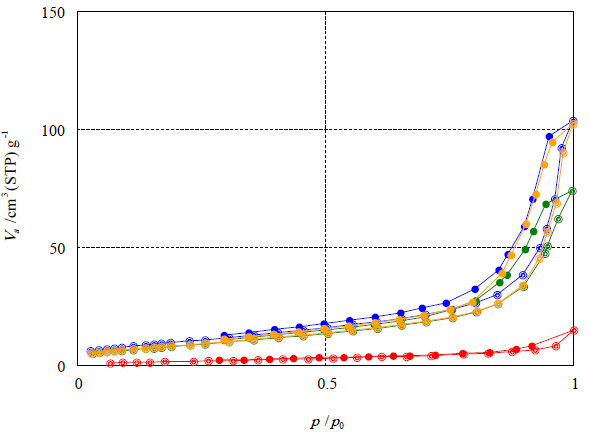 | 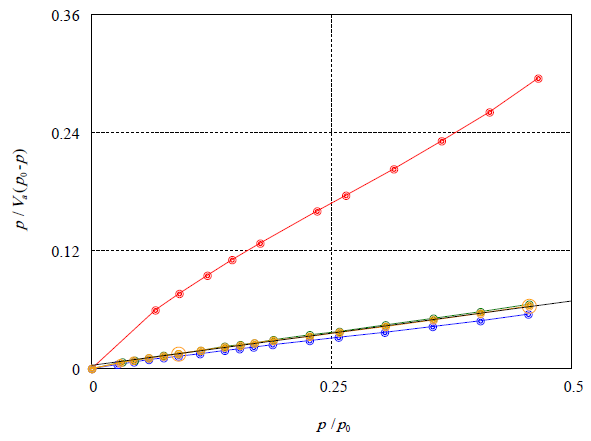 |
| --- | --- |
| (a) | (b) |
| 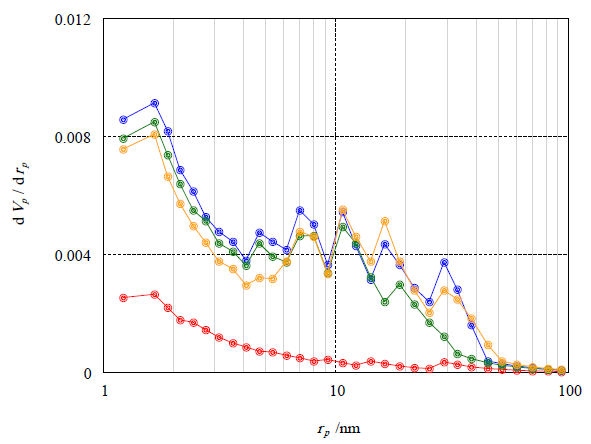 | |
| (c) | |

**Figure S1**. (a) Adsorption–desorption of ZnO (red), 5% Co-ZnO (blue), 10% Co-ZnO (green) and 15% Co-ZnO (yellow); (b) BET analysis; (c) BJH analysis by adsorption.


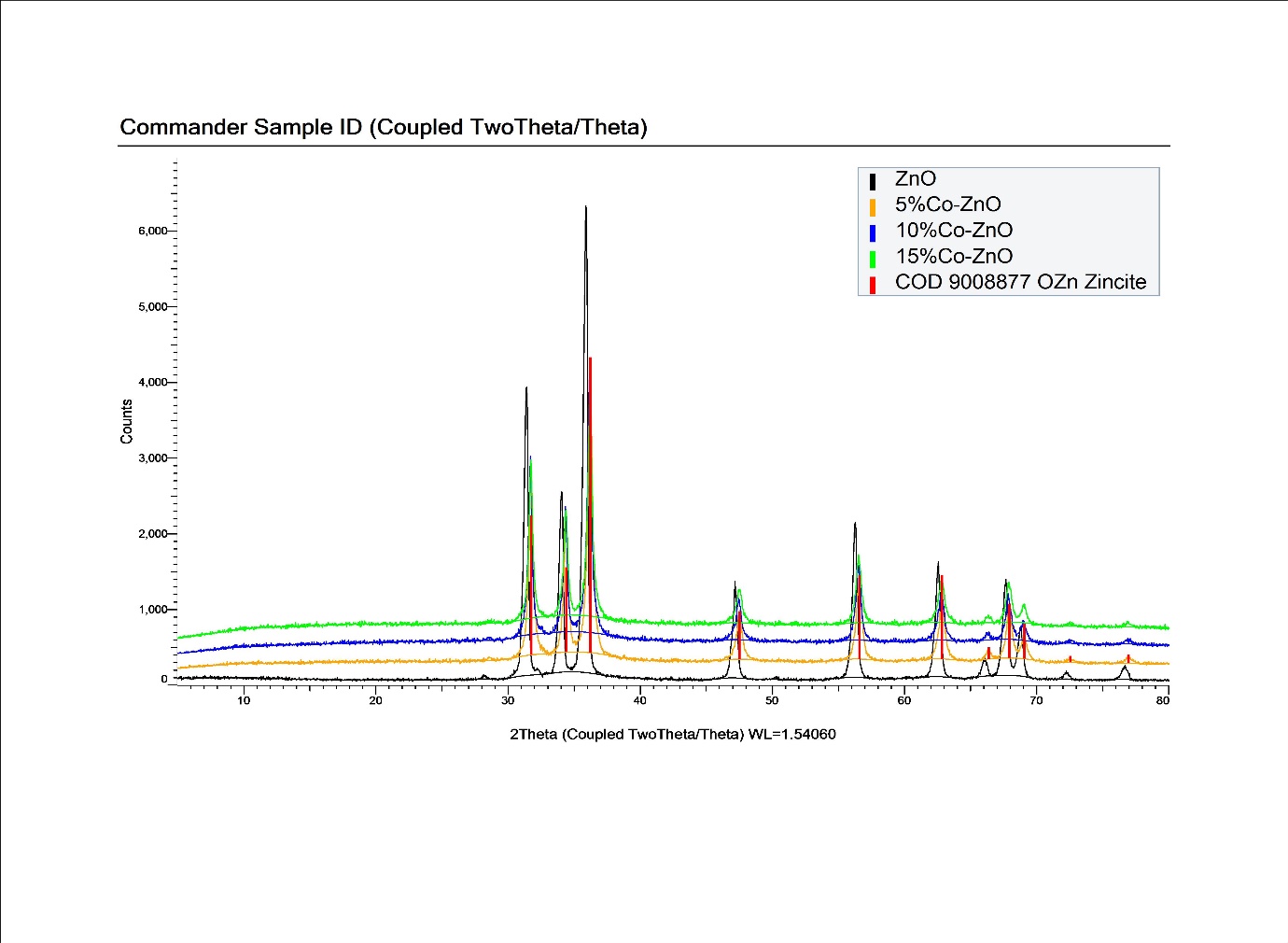


**Figure S2**. X-ray diffraction pattern of ZnO, 5% of Co doped ZnO, 10% of Co doped ZnO and 15 % of Co doped ZnO.

**Table S2**. Crystal size of ZnO NPs and 5, 10, 15% Co-ZnO NPs (nm).

| 2Theta | ZnO-NPs | 2Theta | 5%Co-ZnO | 2Theta | 10%Co-ZnO | 2Theta | 15% Co-ZnO |
| --- | --- | --- | --- | --- | --- | --- | --- |
| 31.38 | 35.64 | 31.75 | 38.85 | 31.705 | 52.70 | 31.735 | 42.88 |
| 34.058 | 36.89 | 34.399 | 39.31 | 34.353 | 51.64 | 34.379 | 39.31 |
| 35.899 | 36.40 | 36.23 | 36.94 | 36.193 | 46.67 | 36.209 | 36.77 |
| 47.193 | 39.23 | 47.521 | 33.26 | 47.473 | 44.80 | 47.499 | 31.36 |
| 56.269 | 39.27 | 56.566 | 34.43 | 56.53 | 48.27 | 56.537 | 36.01 |
| 62.549 | 40.70 | 62.837 | 31.48 | 62.817 | 39.45 | 62.801 | 36.55 |
| 66.086 | 38.82 | 66.373 | 39.56 | 66.307 | 57.10 | 66.388 | 35.24 |
| 67.659 | 38.67 | 67.883 | 36.54 | 67.862 | 48.56 | 67.886 | 36.25 |
| 68.792 | 40.66 | 69.035 | 34.57 | 68.981 | 43.39 | 69.006 | 42.80 |

**Table S3**. A summary of the tested statistical method for CIPF degradation.

| **Source** | **Sum of Squares** | **df** | **Mean Square** | **F-value** | **p-value** |  |
| --- | --- | --- | --- | --- | --- | --- |
| Mean vs Total | 2.177E+05 | 1 | 2.177E+05 |  |  |  |
| Linear vs Mean | 741.29 | 4 | 185.32 | 2.03 | 0.1213 |  |
| 2FI vs Linear | 79.11 | 6 | 13.18 | 0.1135 | 0.9937 |  |
| **Quadratic vs 2FI** | **1304.65** | **4** | **326.16** | **5.42** | **0.0066** | **Suggested** |
| Cubic vs Quadratic | 863.89 | 8 | 107.99 | 19.57 | 0.0004 | Aliased |
| Residual | 38.63 | 7 | 5.52 |  |  |  |
| Total | 2.208E+05 | 30 | 7358.76 |  |  |  |

| 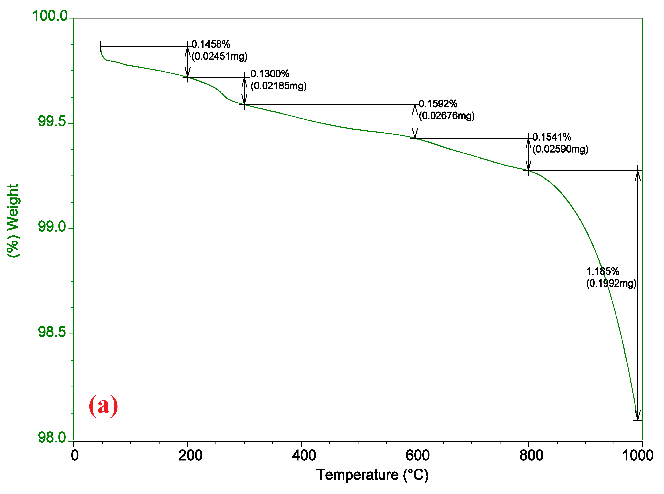 | 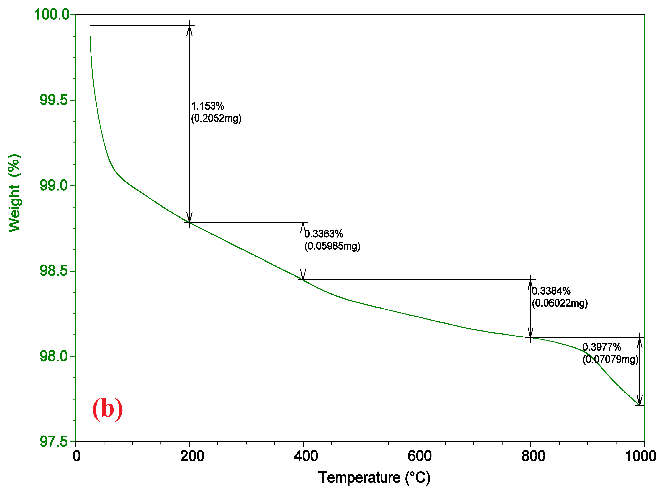 |
| --- | --- |
| 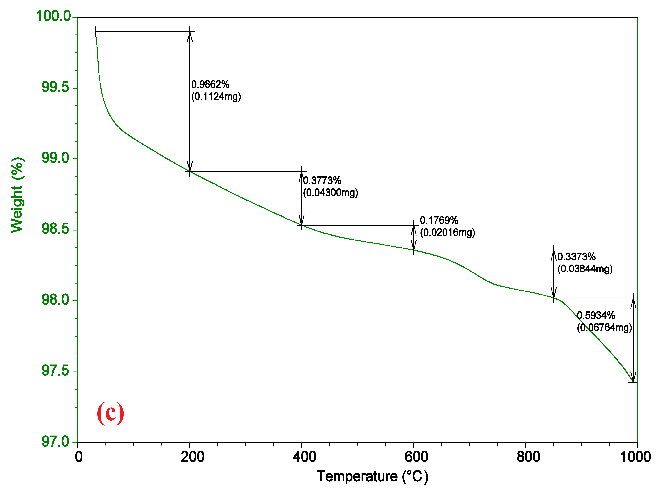 | 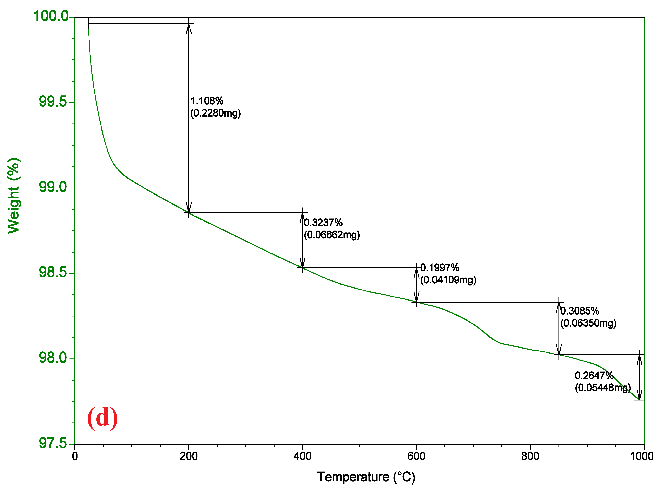 |

**Figure S3**. TGA analyses of a-ZnO,b-5%Co-ZnO,c-10% Co-ZnO and-15% Co-ZnO.

**Table S4.** Comparison of the degradation efficiency of Co-ZnO 10% with other catalysts for Organic contaminants

| **Catalyst Name** | **Dye** | **% Degradation** | **Reference** |
| --- | --- | --- | --- |
| Co3O4 | RhB | 90 % | [1] |
| ZnO | RhB | 95 % | [2] |
| F-MWCNTs/Co–Ti oxide | RhB | 93.35% | [3] |
| C, N-TiO2 | RhB | 94% | [4] |
| NiO | RhB | 80.33% | [5] |
| (Co, Cu)/ ZnO | RhB | 98 % | [6] |
| Co3O4-g-C3N4 (0.1) | MO | 99% | [7] |
| Co-ZnO 5% | MO | 93 | [8] |
| Co-ZnO 10% | CIPF | 99.8 | This study |

The data given in Table S4 shows that the photocatalyst reported in this study is more effective toward photodegradation of CIPF as compared to already reported catalysts.

**Table S5**. Model Summary Statistics

| **Source** | **Std. Dev.** | ***R*²** | **Adjusted *R*²** | **Predicted *R*²** | **PRESS** |  |
| --- | --- | --- | --- | --- | --- | --- |
| Linear | 9.56 | 0.2448 | 0.1240 | -0.1041 | 3342.73 |  |
| 2FI | 10.78 | 0.2710 | -0.1127 | -0.6283 | 4929.77 |  |
| **Quadratic** | **7.76** | **0.7019** | **0.4237** | **-0.7171** | **5198.50** | **Suggested** |
| Cubic | 2.35 | 0.9872 | 0.9471 | -0.8372 | 5562.32 | Aliased |

**Table S6**. Corresponding F-values and P-values and Coefficient estimate for CIPF degradation.

| **Source** | **F-value** | **p-value** | **Coefficient Estimate** | **Standard Error** |
| --- | --- | --- | --- | --- |
| **Model** | 2.52 | 0.0430 | 94.01 | 3.17 |
| A-Catalyst dosage | 0.1725 | 0.6838 | 0.6576 | 1.58 |
| B-Antibiotic dosage | 12.08 | 0.0034 | 5.50 | 1.58 |
| C- Shaking time | 0.0003 | 0.9862 | 0.0279 | 1.58 |
| D-pH | 0.0648 | 0.8026 | -0.4029 | 1.58 |
| AB | 0.0179 | 0.8955 | -0.2591 | 1.94 |
| AC | 0.0007 | 0.9793 | 0.0512 | 1.94 |
| AD | 0.0939 | 0.7635 | 0.5943 | 1.94 |
| BC | 0.3573 | 0.5590 | 1.16 | 1.94 |
| BD | 0.7018 | 0.4153 | -1.62 | 1.94 |
| CD | 0.1432 | 0.7104 | 0.7339 | 1.94 |
| A² | 0.0107 | 0.9188 | -0.1535 | 1.48 |
| B² | 4.00 | 0.0641 | -2.96 | 1.48 |
| C² | 0.9133 | 0.3544 | -1.42 | 1.48 |
| D² | 19.20 | 0.0005 | -6.49 | 1.48 |

| 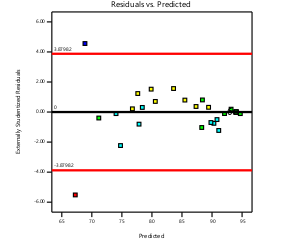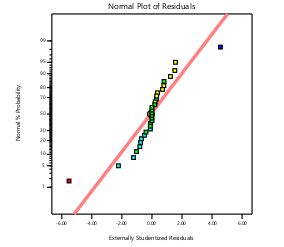)a)  (c) | 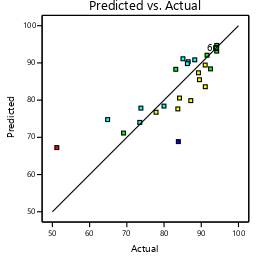(b) |
| --- | --- |

**Figure S4.** The normal probability plot (a), the graphical plot of predicted versus experimental data (b), the graphs of residual versus predicted values (c).

|  |
| --- |

**Figure S5.** Box-Cox plot of the model.

| 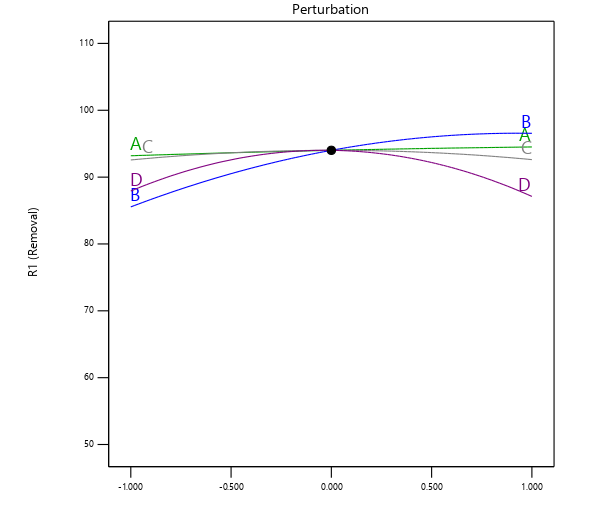 |
| --- |

**Figure S6.** Perturbation plot, catalyst dosage (A), antibiotic concentration (B), Shaking speed (C) and pH value (D).

| 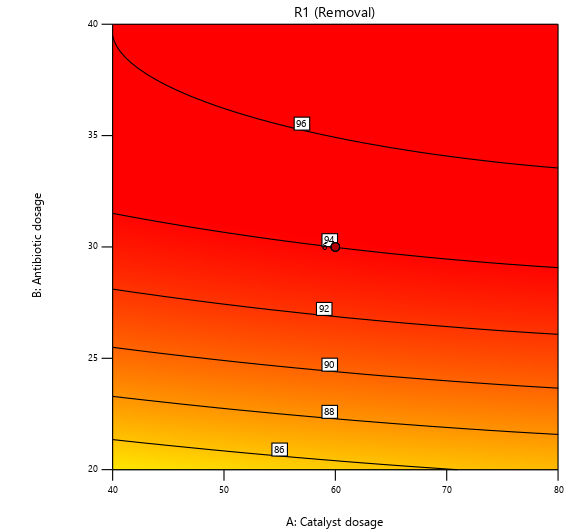 | 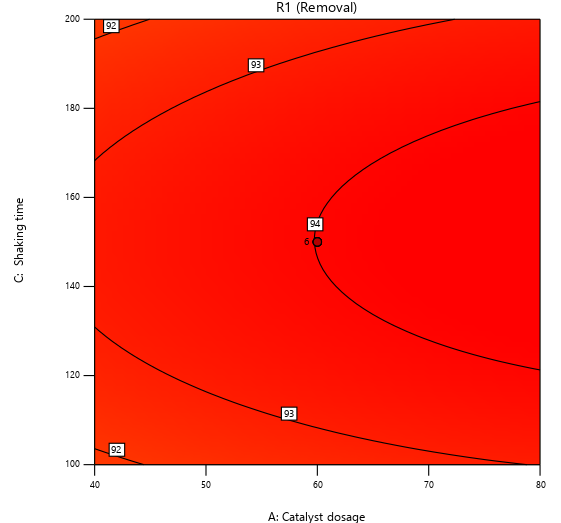 |
| --- | --- |
| 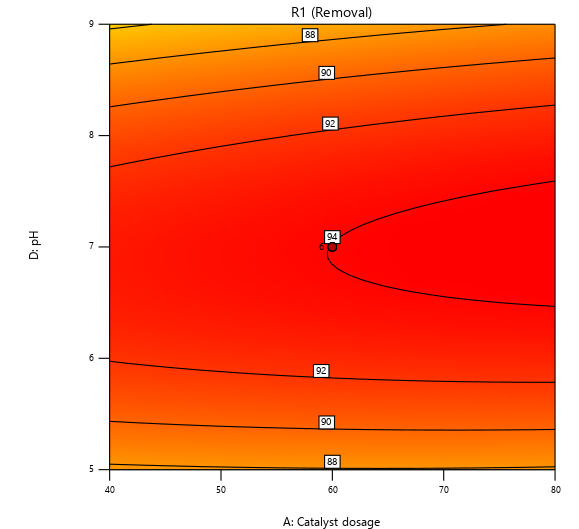 | 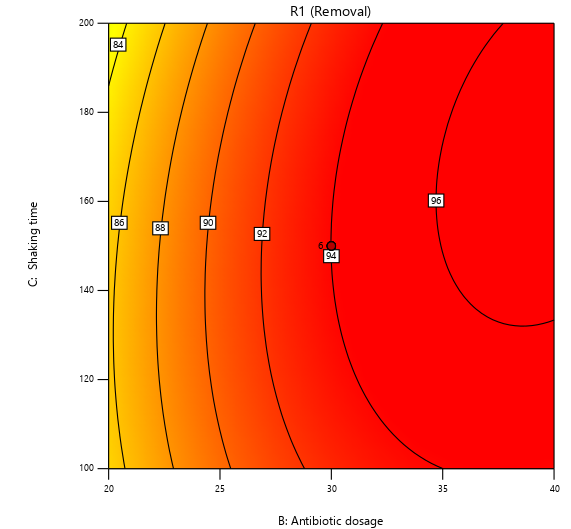 |
| 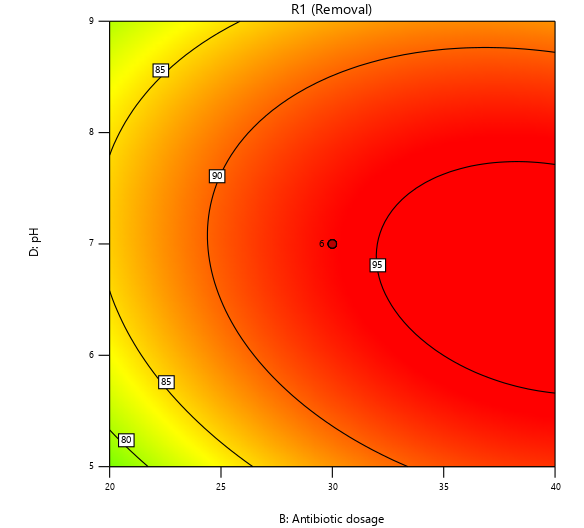 | 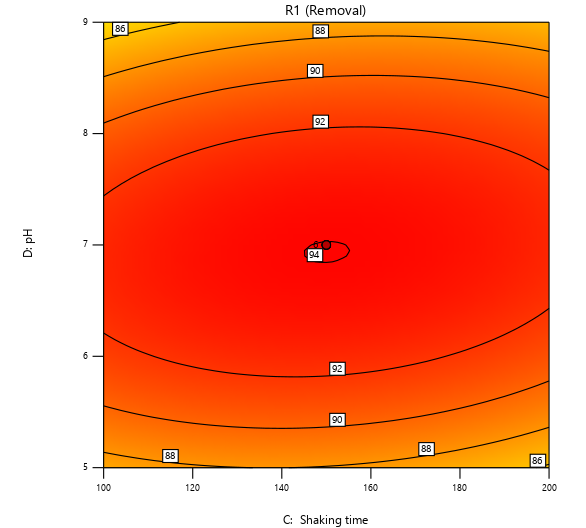 |

**Figure S7.** 2D contours plots for the photodegradation efficiency of CIPF

**References**

[1] Muthukumaran, S., & Gopalakrishnan, R. (2012). Structural, FTIR and photoluminescence studies of Cu doped ZnO nanopowders by co-precipitation method. Optical Materials, 34(11), 1946-1953. <https://doi.org/10.1016/j.optmat.2012.06.004>

[2] Hernandez, A., Maya, L., Sanchez-Mora, E., & Sanchez, E. M. (2007). Sol-gel synthesis, characterization and photocatalytic activity of mixed oxide ZnO-Fe2O3. Journal of Sol-Gel Science and Technology, 42(1), 71-78. <https://doi.org/10.1007/s10971-006-1521-7>

[3] Silva, R. F., & Zaniquelli, M. E. (2002). Morphology of nanometric size particulate aluminium-doped zinc oxide films. Colloids and Surfaces A: Physicochemical and Engineering Aspects, 198, 551-558. <https://doi.org/10.1016/S0927-7757(01)00959-1>

[4] Alkanad, K., Ali, O., GC, S. S., Hezam, A., Bajiri, M. A., & Lokanath, N. K. (2022, March). Highly Efficient Degradation of Organic Compounds via β-Bi2O3Semiconductor Under Visible Illumination. In IOP Conference Series: Materials Science and Engineering (Vol. 1221, No. 1, p. 012040). IOP Publishing. <https://doi.org/10.1088/1757-899X/1221/1/012040>

[5] Rahman, Q. I., Ahmad, M., Misra, S. K., & Lohani, M. (2013). Effective photocatalytic degradation of rhodamine B dye by ZnO nanoparticles. Materials Letters, 91, 170-174. <https://doi.org/10.1016/j.matlet.2012.09.044>

[6] Balasubramani, K. and Ayyadurai, S., 2022. Synthesis of Co and Cu codoped ZnO nanoparticles by citrate gel combustion method: Photocatalytic and antimicrobial activity. *Journal of Water and Environmental Nanotechnology*, *7*(2), pp.143-154.

[7] Han, C.; Ge, L.; Chen, C.; Li, Y.; Xiao, X.; Zhang, Y.; Guo, L. Novel Visible Light Induced Co3O4-g-C3N4 Heterojunction Photocatalysts for Efficient Degradation of Methyl Orange. Appl. Catal., B 2014, 147, 546−553.

[8] Adeel, M., Saeed, M., Khan, I., Muneer, M. and Akram, N., 2021. Synthesis and characterization of Co–ZnO and evaluation of its photocatalytic activity for photodegradation of methyl orange. *ACS omega*, *6*(2), pp.1426-1435.
